# A phase I study of niclosamide in combination with enzalutamide in men with castration-resistant prostate cancer

**DOI:** 10.1371/journal.pone.0198389

**Published:** 2018-06-01

**Authors:** Michael T. Schweizer, Kathleen Haugk, Jožefa S. McKiernan, Roman Gulati, Heather H. Cheng, Jessica L. Maes, Ruth F. Dumpit, Peter S. Nelson, Bruce Montgomery, Jeannine S. McCune, Stephen R. Plymate, Evan Y. Yu

**Affiliations:** 1 Department of Medicine, University of Washington, Seattle, Washington, United States of America; 2 Clinical Research Division, Fred Hutchinson Cancer Research Center, Seattle, Washington, United States of America; 3 Veterans Affairs Puget Sound Health Care System, University of Washington, Seattle, Washington, United States of America; 4 Department of Population Sciences, City of Hope Comprehensive Cancer Center, Duarte, California, United States of America; 5 Division of Public Health Sciences, Fred Hutchinson Cancer Research Center, Seattle, WA; 6 Seattle Cancer Care Alliance, Seattle, Washington, United States of America; 7 Division of Human Biology, Fred Hutchinson Cancer Research Center, Seattle, Washington, United States of America; National Cancer Centre Singapore, SINGAPORE

## Abstract

**Background:**

Niclosamide, an FDA-approved anti-helminthic drug, has activity in preclinical models of castration-resistant prostate cancer (CRPC). Potential mechanisms of action include degrading constitutively active androgen receptor splice variants (AR-Vs) or inhibiting other drug-resistance pathways (e.g., Wnt-signaling). Published pharmacokinetics data suggests that niclosamide has poor oral bioavailability, potentially limiting its use as a cancer drug. Therefore, we launched a Phase I study testing oral niclosamide in combination with enzalutamide, for longer and at higher doses than those used to treat helminthic infections.

**Methods:**

We conducted a Phase I dose-escalation study testing oral niclosamide plus standard-dose enzalutamide in men with metastatic CRPC previously treated with abiraterone. Niclosamide was given three-times-daily (TID) at the following dose-levels: 500, 1000 or 1500mg. The primary objective was to assess safety. Secondary objectives, included measuring AR-V expression from circulating tumor cells (CTCs) using the AdnaTest assay, evaluating PSA changes and determining niclosamide’s pharmacokinetic profile.

**Results:**

20 patients screened and 5 enrolled after passing all screening procedures. 13(65%) patients had detectable CTCs, but only one was AR-V+. There were no dose-limiting toxicities (DLTs) in 3 patients on the 500mg TID cohort; however, both (N = 2) subjects on the 1000mg TID cohort experienced DLTs (prolonged grade 3 nausea, vomiting, diarrhea; and colitis). The maximum plasma concentration ranged from 35.7–82 ng/mL and was not consistently above the minimum effective concentration in preclinical studies. There were no PSA declines in any enrolled subject. Because plasma concentrations at the maximum tolerated dose (500mg TID) were not consistently above the expected therapeutic threshold, the Data Safety Monitoring Board closed the study for futility.

**Conclusions:**

Oral niclosamide could not be escalated above 500mg TID, and plasma concentrations were not consistently above the threshold shown to inhibit growth in CRPC models. Oral niclosamide is not a viable compound for repurposing as a CRPC treatment.

**Clinical trial registry:**

Clinicaltrials.gov: NCT02532114

## Introduction

Nearly 30,000 American men die as a result of their prostate cancer each year[1]. Since the 1940s, the treatment of advanced prostate cancer has focused almost exclusively on inhibiting the androgen receptor (AR)-signaling program[2]. Indeed, over the past decade it has been discovered that even in men with castration-resistant prostate cancer (CRPC)–a clinical state defined by disease progression in spite of medical or surgical castration (i.e., androgen deprivation therapy)–AR remains the primary driver[3, 4]. This realization has led to the further exploration of the AR-signaling axis as a therapeutic target in men with metastatic CRPC (mCRPC), and led to the development of effective new AR-directed agents like abiraterone and enzalutamide, which inhibit AR-signaling through disrupting the ligand-receptor interaction (abiraterone through ligand depletion and enzalutamide through receptor antagonism)[5–8]. These agents are unfortunately not curative, and resistance typically occurs in 1–2 years.

Several mechanisms of resistance to next-generation AR-directed therapies have been described, including: i) activation of canonical AR-signaling through *AR* amplification, AR overexpression and/or maintenance of intratumoral androgens; ii) AR-signaling activation via feedback pathways (e.g. AKT/mTOR/Pi3K, NF-κB, Wnt/β-catenin); and iii) activation of the AR program via mutations (e.g. *AR* ligand binding domain mutation) or AR substitutions (e.g. AR splice variants; Glucocorticoid Receptor-signaling)[9–22]. Of these mechanisms, the emergence of alternatively spliced AR variants (AR-Vs), which maintain constitutive activity in spite of lacking the AR ligand-binding domain, has received substantial attention. Inhibiting AR-V activity has been shown to be an effective strategy in preclinical models and the emergence of AR-V7, the most prevalent AR-V, has been associated with a lack of response to abiraterone and enzalutamide[9, 13, 23]. While the emergence of AR-Vs provides an elegant biologic rationale for why drugs that interfere with the AR-ligand interaction may not be effective, it remains unclear whether AR-V expression is a driver of disease progression or merely a reflection that a larger resistance program has been activated[13, 23–27].

Given that all the approved AR-signaling inhibitors work by preventing ligand-AR interaction, an agent that can effectively disrupt AR-V signaling, or inhibit other relevant resistance pathways, would be an invaluable therapeutic option for men with multi-drug resistant mCRPC. Niclosamide, an FDA-approved anti-helminthic drug, has been shown in several preclinical models of CRPC to be a potent anti-neoplastic agent. It results in decreased cell proliferation across multiple cell lines, with a reported IC50 of 330 ng/mL[28]. Most studies have tested concentrations below the IC50, with decreased cellular viability occurring at concentrations from 81.8 to 327 ng/mL. Mechanistic studies have shown that niclosamide may exert its effect through degrading AR-Vs or through inhibiting other pathways, including AKT/mTOR/Pi3K, NF-κB, and Wnt-signaling, which are implicated in prostate cancer resistance and progression[17, 28–32]. Interestingly, niclosamide does not appear to have an effect on full-length AR expression, providing a rationale for testing this drug in combination with next-generation AR-signaling inhibitors (i.e., abiraterone or enzalutamide).

Published data regarding the pharmacokinetics (PK) of niclosamide suggest that it has poor oral bioavailability, potentially limiting its use as a cancer drug. The standard oral dose of niclosamide used to treat anti-helminthic infections in adults is 2000 mg daily for 1–7 days, and in a cohort of healthy male and female volunteers administered a single 2000 mg oral dose of carbonyl-^14^C-labeled niclosamide, the maximum serum concentration of niclosamide (C_max_) attained was estimated to be between 250 to 6000 ng/mL[33–35]. These data suggest that the standard anti-helminthic dose of niclosamide may not result in serum concentrations that are consistently in the therapeutic range shown to inhibit prostate cancer growth. On the basis of the aforementioned data, we launched a Phase I study to test oral niclosamide in combination with enzalutamide in men with mCRPC who have progressed on abiraterone. Our primary goal was to evaluate the safety, tolerability, and pharmacokinetics of niclosamide administered, in combination with enzalutamide, for longer (i.e., >7 days) and at higher doses (i.e., >2000 mg daily) than those used to treat helminthic infections.

## Methods

### Study design

This was an open label, Phase I dose-escalation study testing high-dose niclosamide in combination with the FDA-approved dose of enzalutamide (160 mg by mouth daily) [clinicaltrials.gov: NCT02532114]. This study was approved by the University of Washington/Fred Hutchinson Cancer Research Center Institutional Review Board and written informed consent was obtained from all enrolled subjects. Study participants were recruited through medical oncology clinics at the University of Washington and Seattle Cancer Care Alliance (both in Seattle, WA), and the study was open for recruitment from October 2015 to November 2017, with all patient follow up completing in December 2017. Niclosamide was given three-times-daily (TID) by mouth (PO) for four weeks at one of the following dosing cohorts: 500, 1000, or 1500 mg. All patients were required to have mCRPC (i.e., disease progression in spite of serum testosterone ≤50 ng/dL) and had received prior abiraterone. Patients were originally required to have detectable AR-V transcripts in circulating tumor cells (CTC) as determined using the AdnaTest assay[9]. However, due to very few AR-V+ patients in the initial screen, and recognition that niclosamide may exert antineoplastic effect through AR-V+ independent pathways, the protocol was modified to remove this eligibility criteria. Given that niclosamide has been shown to impair multiple mechanisms of resistance to enzalutamide, patients were permitted to start enzalutamide prior to the addition of niclosamide, and evidence of disease progression on enzalutamide was not exclusionary[17, 28–32]. Enrolled subjects were also required to have a creatinine clearance >30 mL/min, Eastern Cooperative Oncology Group (ECOG) performance status ≥2 and no signs of severe hepatic impairment (i.e., Child-Pugh Class C). The primary objective was to assess safety. Key secondary objectives were to assess the pharmacokinetic profile of niclosamide, evaluate prostate-specific antigen (PSA) changes following 4 weeks of niclosamide, and to assess pharmacodynamic effects on CTCs.

### Pharmacokinetics

The pharmacokinetic samples were quantitated for plasma niclosamide concentrations using a modification of previously published methods[36]. Blood for pharmacokinetic analyses were drawn at the following time points after the first dose: 0.5 hr, 1 hr, 1.5 hr, 2 hr, 3 hr, 4 hr, 6 hr, 8 hr and on Day 15. In brief, patient plasma (25 μL) was mixed with the internal standard solution (20 μL containing 50 ng niclosamide ^13^C_6_ in methanol) and methanol (200 μL) in a 0.5 mL snap-cap micro-centrifuge tube. After vortexing, the samples were centrifuged at 20000 x G for 10 minutes at 4°C. The supernatant was transferred to a 96-well plate and 2 μL were injected on the liquid chromatography mass spectrometry (LCMS) system. The LCMS system was an ultrapressure liquid chromatography (Agilent (Santa Clara, CA) 1290 series) coupled to an Agilent G6410B triple-quadrupole mass spectrometer. The column was an Agilent Zorbax SB-C18 2.1 mm x 150 mm x 5μ maintained at 45°C. Mobile phase A was 10 mM ammonium formate (pH = 3) and mobile phase B was acetonitrile in the ratio of 35% A: 65% B at 0.4 mL/min.

The mass spectrometer was operated in the ESI-negative mode with the following transitions: 324.8→170.9 *m/z* (niclosamide) and 330.9→176.9 *m/z* (niclosamide ^13^C_6_).

A ten-point calibration curve was created by spiking blank plasma with niclosamide to make standards in the range of 40.8 to 8160 ng/mL and processing the standards identically to samples. Calibration curves were based upon the height ratio of the niclosamide to the internal standard (niclosamide ^13^C_6_) and were fitted using a polynomial fit with 1/x weighting. The correlation coefficient was used to evaluate the linearity of the calibration curves and was >0.99 in all experiments. The limit of quantitation was 40.8 ng/mL (CV = 11.3%; accuracy 104.9%) and the limit of detection was 16.3 ng/mL, with a signal to noise ratio of 53.75.

### Splice variant determination

The presence of AR-V transcripts were determined from CTCs using qRT-PCR and primers designed to detect AR-V7 and AR-V567es (i.e., exon 5, 6 and 7 deleted AR-V) mRNA. The AdnaTest ProstateCancerSelect/ProstateCancerDetect assay (AdnaGen, Langenhagen, Germany) and methods similar to those described by Antonarakis and colleagues were used[9]. AR-V PCR product visualization was on 2% agarose gel. All variant PCR products were evaluated for size and concentration using the QIAxcel Advanced System (QIAGEN, Inc; Germantown, MD, USA).

### Statistical considerations

We utilized the continual reassessment method (CRM) to evaluate dose-related toxicities and to determine the recommended Phase II dose[37]. We targeted a maximum of 30% of patients incurring dose-limiting toxicities (DLT) up to 30 days after the final (day-28) dose. Dose escalation and de-escalation were dictated by the CRM and based on posterior probabilities determined by: 1) the assumed dose-toxicity model, which was a 1-parameter power model with a Gamma(1,1) prior distribution; 2) assumed prior probabilities of DLTs of 5%, 10%, and 15% for dose levels 500, 1000 and 1500 mg PO TID, respectively; 3) a target DLT rate of ≤30%; and 4) accumulating toxicity data. Adverse events (AEs) were documented by incidence and their severity was graded according to the National Cancer Institute–Common Terminology Criteria for Adverse Events (CTCAE) version 4.0. The pharmacokinetic data underwent non-compartmental analysis using Phoenix WinNonlin version 8.0 (Certara USA, Inc; Princeton, NJ), and key pharmacokinetic parameters (e.g., C_max_, C_min_, C_ss_ and t_1/2_) were extracted.

## Results

### Patients

**Fig 1:**
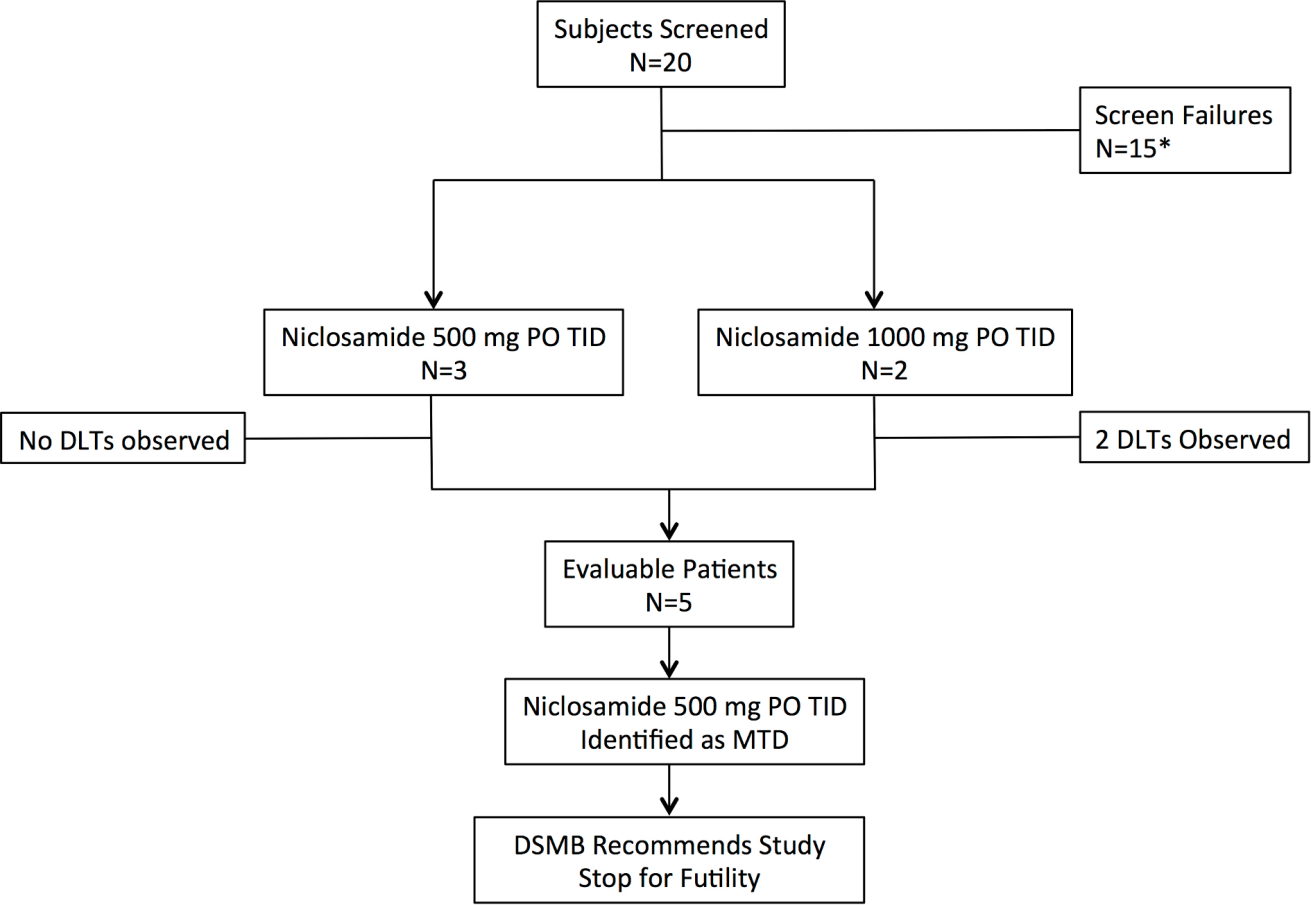
Study flow diagram. *All screen failures were due to undetectable androgen receptor splice variants, which were mandated to be present under protocol version 1. Protocol version 2 removed this criterion. MTD, maximum tolerated dose; TID, three times daily; PO, by mouth; DSMB, data safety monitoring committee.

From December 2015 to October 2017, 20 patients were screened and 5 passed all screening procedures and enrolled onto the study (Fig 1). The 15 screen failures occurred under protocol version 1 and were a consequence of undetectable AR-Vs. All patients previously progressed on enzalutamide, with 18/20 patients demonstrating a rising PSA on enzalutamide at the time of screening. Baseline patient demographics for those who enrolled in the trial are presented in Table 1. Two enrolled patients had previously documented AR-Vs as determined from prior transcript profiling studies; however, only one of these patients was AR-V positive (AR-V56) at the time of study enrollment. Of the 18 patients without prior evidence of an AR-V, none were found to be AR-V+. All patients were positive for actin (positive control) and 13/20 had detectable CTCs as indicated by the presence of at least one additional tumor-associated transcript (i.e., full-length AR, PSA, and/or prostate-specific membrane antigen) using the AdnaTest (Table 2). There was no evidence for declining PSA across any of the dose cohorts (Table 3).

**Table 1:** Baseline characteristics of study population at time of enrollment. Patient characteristics at the time of screening. *PSA was previously rising on enzalutamide but began falling after palliative radiation to a painful bone metastasis, which was administered just prior to initiating niclosamide.

					Sites of disease			
Pt#	Age	PSA (ng/mL)	Hemoglobin	Total Gleason Score	Bone	Lymph nodes	Visceral	Actively progressing on enzalutamide?
1	84	70.77	10.6	9	Yes	Yes	No	Yes
17	75	63.66	13.6	9	Yes	No	No	Yes
18	71	100.99	11.7	9	Yes	Yes	Yes	Yes
19	68	1492.36	9.4	7	Yes	No	No	Yes
20	60	31.02	8.2	9	Yes	Yes	No	No*

**Table 2:** AR splice variant detection using AdnaTest.

Pt#	Timepoint	Actin	PSA	PSMA	AR-FL	AR-V7	ARv567*	ARv56*
**Screened and treated**								
1	Screening	+			+		+	+
1	Day 29	+			+	+	+	+
17	Screening	+						
17	Day 29	+						
18	Screening	+	+	+	+			
18	Day 29	+			+			
19	Screening	+	+	+	+			
19	Day 29	+	+	+	+			
20	Screening	+						
20	Day 21**	+			+			
**Only screened**								
2	Screening	+			+			
3	Screening	+			+			
4	Screening	+	+		+			
5	Screening	+						
6	Screening	+						
7	Screening	+			+			
8	Screening	+						
9	Screening	+			+			
10	Screening	+						
11	Screening	+			+			
12	Screening	+			+			
13	Screening	+	+		+			
14	Screening	+						
15	Screening	+	+		+			
16	Screening	+			+			

Tumor-associated transcripts (PCR fragment) are detected by visualization on 2% agarose gel (+/-). Variant products with a concentration greater than or equal to 100 ng/mL are considered positive.

*ARv567 and ARv56 are indistinguishable on agarose gel. The presence of these variants was determined through sequencing the respective bands.

**Patient taken off study early secondary to a dose-limiting toxicity.

**Table 3:** Summary of on-study PSA changes and adverse events.

		PSA (ng/mL)			Adverse Events Possibly/Probably Related to Niclosamide							
Pt#	Niclosamide cohort	Day 1	Day 29	PSA Change (%)	Nausea (Grade)	Anorexia (Grade)	Vomiting (Grade)	Diarrhea (Grade)	Weight loss (Grade)	Lipase elevation (Grade)	Colitis (Grade)	Abdominal pain (Grade)
1	500 mg PO TID	70.77	78.54	9.9%	—	—	—	—	—	—	—	—
17	500 mg PO TID	63.66	114.77	44.5%	—	—	—	—	—	—	—	—
18	500 mg PO TID	100.99	188.8	46.5%	1	1	—	—	1	—	—	—
19	1000 mg PO TID	1492.36	3009.49	101.7%	3*	—	3	3	—	2	—	—
20	1000 mg PO TID	31.02	—	—	2	1	—	3	—	—	3**	3

*Constituted a dose-limiting toxicity given that these AEs lasted >72 hours.

**Dose limiting toxicity.

### Adverse events

Niclosamide was well tolerated in combination with enzalutamide in the first dose cohort (i.e., niclosamide 500 mg PO TID), and only one out of three patients at this dose level experienced any toxicities deemed at least possibly related to niclosamide (Grade 1 nausea, anorexia and weight loss). Both patients treated at the second dose level (i.e., niclosamide 1000 mg PO TID) experienced dose-limiting toxicities. Patient #19 had Grade 3 nausea, vomiting, and diarrhea lasting >72 hours. Symptoms began on Day 26 of treatment, and he discontinued the study drugs that day. He was ultimately hospitalized between Days 32 to 40 as a result of this AE. This patient also experienced a Grade 2 lipase elevation without other signs of pancreatitis, which was felt to be possibly related to niclosamide. At the time of discharge he had increased oral intake without nausea or vomiting. Patient #20 had Grade 3 colitis, abdominal pain and diarrhea, with the colitis constituting a DLT. Symptoms began on Day 8 of treatment and abdominal CT on Day 9 revealed evidence of colitis. He was subsequently admitted to the hospital between Days 9 to 12 where he received aggressive supportive care (i.e. IV hydration and antibiotics). This AE had returned to Grade 1 at the time of discharge. It should be noted that this patient had received an immune checkpoint inhibitor as part of a clinical trial immediately prior to enrolling onto this study, and a colonoscopy with biopsy was performed to evaluate for delayed onset immune-mediated colitis. Pathologic findings from this biopsy were not consistent with an immune-mediated adverse event, however, and it was felt that niclosamide was likely the causative factor. A summary of all AEs deemed at least possibly related to niclosamide are provided in Table 3.

### Pharmacokinetic results

Considering the enthusiasm for developing niclosamide for AR-V+ mCRPC, we chose to include all pharmacokinetic data. Pharmacokinetic data were obtained after the first dose in all patients and after the morning dose on the 15^th^ day of treatment in four patients (Table 4). It should be noted that the timing of the Day 15 blood draw in relation to niclosamide dosing was not recorded, and therefore this timepoint was excluded from the pharmacokinetic analyses. The maximum observed plasma concentration ranged from 35.7 to 182 ng/mL, which, for most patients, was below the minimum effective concentration in preclinical studies[28, 29, 38]. We also estimated the total area under the curve (AUC_0-∞_) after the first dose, which should be evaluated with caution in those participants whose AUC_0-∞_ was over 20% extrapolated. The apparent oral clearance of niclosamide, which is clearance divided by the oral bioavailability (i.e., CL/F), ranged from 9.09 to 37.8 L/hr per kg of ideal body weight. Because niclosamide plasma concentrations in the maximal tolerated dosing cohort (i.e., 500 mg TID) were below those expected to exert an anti-tumor effect, the study was closed for futility.

**Table 4:** Pharmacokinetic results summary.

Pt#	Dose	T_max_	C_max_	C_max_ /dose	T_min_	C_min_^a^	T_1/2_	First dose AUC_0-τ_	First dose AUC_0-∞_^a^	% of AUC_0-∞_ extrapolated^a^	Apparent oral clearance^b^
(unit)	(mg)	(hr)	(ng/mL)	(ng/mL per mg dose)	(hr)	(ng/mL)	(hr)	(ng×hr /mL)	(ng×hr/mL)	%	(L/hr per kg of IBW^c^)
**1**^**d**^	500	6	182	0.363	4	13.3	1.27	692	942	26.6	9.09
**17**	500	2	35.7	0.072	8	6.92	2.24	155	180	13.7	37.8
**18**	500	1	88.3	0.177	8	7.42	3.86	202	243	17.2	26.5
**19**	1000	1	182	0.182	8	45.5	5.61	676	1060	36.0	12.2
**20**	1000	6	149	0.149	8	90.0	2.75	629	986	36.2	14.9

^a^C_last_/elimination rate constant was used to estimate the AUC from the end of the dosing interval to time infinity (∞)

^b^clearance divided by fraction absorbed

^c^IBW = 50 kg + 2.3 kg per inch over 5 feet.

^d^Patient had an aberrant pharmacokinetic profile with a second peak at 6h. Evaluate with caution.

## Discussion

Our main findings are: 1) that niclosamide doses could not be escalated above 500 mg PO TID because of toxicity; 2) niclosamide is not a viable oral compound for repurposing as a mCRPC treatment because the dosing cohort with acceptable toxicity (i.e., 500 mg PO TID) does not consistently yield concentrations above those shown to inhibit tumor growth in mCRPC models; and 3) niclosamide pharmacokinetics had moderate variability with our C_max_ data agreeing with previous reports[17, 28–32]. Niclosamide has impressive preclinical activity across a range of malignancies, and is currently being investigated in several early phase clinical trials targeting patients with mCRPC as well as colorectal cancer [clinicaltrials.gov: NCT02687009, NCT03123978, NCT02519582, NCT02807805][17, 29–32]. Because niclosamide has been reported to have poor oral bioavailability, we sought to test higher doses of niclosamide than those used to treat helminth infections as a means to increase plasma niclosamide concentrations[35]. Ultimately, we were unable to reach a dose that resulted in plasma niclosamide concentrations predicted to exert an anti-tumor effect.

Prior preclinical studies testing niclosamide reported decreased DU145 proliferation at a half-maximal inhibitory concentration (IC50) of 330 ng/mL, and niclosamide’s anti-tumor effects are apparent across a range of prostate cancer models, with decreased cellular viability documented at concentrations from 81.8 to 327 ng/mL. Liu and colleagues have published a series of papers examining the effects of niclosamide in abiraterone and/or enzalutamide-resistant models, including several with AR-V7 expression[29, 38, 39]. They found that niclosamide concentrations ≥163.5 ng/mL consistently inhibited cell growth and that some AR-V7+ cell lines were inhibited by concentrations as low as 81.8 ng/mL. For instance, enzalutamide-resistant, AR-V7+ C4-2B cells were growth inhibited when co-cultured with 81.8 ng/mL of niclosamide; however, AR-V7+ CWR22Rv1 cells did not demonstrate significant growth inhibition when exposed to this concentration of niclosamide[29]. Similarly, enzalutamide-resistant LNCaP cells that had been engineered to maintain Stat3 activation were growth inhibited only when exposed to niclosamide concentrations ≥163.5 ng/mL[39]. It is worth noting that both of these studies demonstrated a growth inhibitory effect with lower concentrations of niclosamide when cells were treated concurrently with enzalutamide. On the basis of these data, we concluded that niclosamide plasma concentrations below 163.5 ng/mL were unlikely to exert a clinically meaningful effect. Overall, we observed one of three patients in the 500 mg PO TID cohort and one of two patients in the 1000 mg PO TID cohort achieve a C_max_ ≥163.5 ng/mL. There was no evidence of clinical activity in any patient enrolled and toxicity prevented dose escalation above the 500 mg PO TID cohort. Therefore, the Data Safety Monitoring Board for the trial recommended that the trial terminate prematurely for futility.

This study was originally designed to include AR-V positivity as a novel integral biomarker given that: 1) AR-V7 has been hypothesized to promote resistance to drugs that interfere with the AR-ligand/AR interaction (i.e., abiraterone and enzalutamide); 2) niclosamide has been shown to be effective in preclinical models of AR-V+ CRPC; and 3) prior studies documented AR-V positivity in >45% of mCRPC patients post-abiraterone and/or enzalutamide[9, 29, 38, 40, 41]. However, the proportion of AR-V+ patients in our study fell well short of the 45% prevalence mark, with only 5% of screened subjects testing positive using the AdnaTest[9, 40]. Because of low AR-V detection rates, we ultimately abandoned AR-V positivity as an inclusion criterion. It is notable that our experience with AR-V detection closely mirrors that of the ARMOR 3 Trial, a Phase III trial testing galeterone (an oral AR-signaling inhibitor that has also been shown to degrade AR-Vs), which documented 8% AR-V detection in men with treatment-naïve mCRPC[41, 42]. This study, as well as the ARMOR 3 Trial, highlights the importance of having assays that have been validated across institutions and have undergone rigorous pre-study confirmatory testing.

The small sample size of this trial represents its primary limitation, and while relatively few patients were treated per protocol, our data still represent the largest cohort of niclosamide-treated patients with contemporary safety and pharmacokinetic data. Prior conclusions regarding the pharmacokinetic profile of niclosamide all trace back to one internal Bayer document from 1971 in which an undisclosed number of healthy volunteers received a single dose of ^14^C-labeled niclosamide[35]. Ultimately, the plasma niclosamide concentrations observed at the maximum tolerated dose (i.e., niclosamide 500 mg PO TID) were not consistently in the range shown to have activity in preclinical models of abiraterone and enzalutamide-resistant CRPC, and toxicity prevented us from escalating to a therapeutic dose. It is important to bear in mind, however, that because we did not evaluate enzalutamide pharmacokinetics, we are unable to exclude the possibility that there was a drug-drug interaction between enzalutamide and niclosamide. In addition, there was insufficient data to characterize niclosamide’s steady state pharmacokinetics and it remains possible that niclosamide may accumulate over time or undergo more rapid clearance.

Overall, we conclude that the development of the current oral formulation of niclosamide as a cancer therapy should not be pursued. Attention should be turned to developing niclosamide analogs with improved oral bioavailability and enhanced antitumor effects.

## Supporting information

S1 TREND Checklist TREND statement checklist. (PDF)

S1 Protocol Clinical protocol. (PDF)
