# Correction: A phase I study of niclosamide in combination with enzalutamide in men with castration-resistant prostate cancer

**DOI:** 10.1371/journal.pone.0202709

**Published:** 2018-08-15

**Authors:** Michael T. Schweizer, Kathleen Haugk, Jožefa S. McKiernan, Roman Gulati, Heather H. Cheng, Jessica L. Maes, Ruth F. Dumpit, Peter S. Nelson, Bruce Montgomery, Jeannine S. McCune, Stephen R. Plymate, Evan Y. Yu

There is an error in the maximum plasma concentration of niclosamide reported in the “Results” section of the Abstract. The correct concentration range is: 35.7 to 182 ng/mL. This range is correctly reported in the “Pharmacokinetic results” section of the article as well as in Table 4.
