# Supplementary material for: A phase I study of niclosamide in combination with enzalutamide in men with castration-resistant prostate cancer
Source: PLoS One. 2018 Jun 1;13(6):e0198389. doi: 10.1371/journal.pone.0198389 (PMC5983471; doi:10.1371/journal.pone.0198389)
Supplement: S1 Protocol — (PDF) [file pone.0198389.s002.pdf]

IRB Approved
Document Release Date: 07/26/2017

A Phase I Study of Niclosamide in Combination with Enzalutamide in Men with Castration-Resistant Prostate Cancer

University of Washington/Fred Hutchinson Cancer Research Center

Protocol Number: CC9390

IND Number: 126412

Protocol Version Number: 3

February 27, 2017

Principal Investigator:

Michael Schweizer, MD
University of Washington
Email: schweize@uw.edu

Statistician:

Roman Gulati, MS
Fred Hutchinson Cancer Research Center
Email: rgulati@fhcrc.org

Title: A Phase I Study of Niclosamide in Combination with Enzalutamide in Men with Castration-Resistant Prostate Cancer

Objectives: To determine the safety and tolerability of high-dose niclosamide combined with enzalutamide in men with castration-resistant prostate cancer (CRPC) that has progressed on abiraterone.

Study Design: Open label, single-institution, Phase I dose-escalation study designed to determine the safety and tolerability of TID niclosamide dosing when given in combination with enzalutamide.

Primary Location: Seattle Cancer Care Alliance

Timeline: This study is planned to complete enrollment in 18 months, with an additional 3 months of follow up following accrual of the last patient.

Concept Rationale: The observation that androgen receptor (AR) regulated genes (e.g. PSA) remain expressed in a castrate state led to the further exploration of the AR-signaling axis as a therapeutic target in men with castration resistant prostate cancer (CRPC). These observations have led to the development of effective new AR-directed agents like abiraterone (Abi) and enzalutamide (Enza) which inhibit AR-signaling in men with CRPC through disrupting the ligand-AR interaction (Abi through ligand depletion and Enza through receptor antagonism).[1-4] These agents are unfortunately not curative, however, with resistance typically occurring within one year. A number of mechanisms have been described by which resistance occurs to these next-generation AR-directed agents, including: alterations leading to persistent canonical AR-signaling (e.g. AR amplification/overexpression, elucidations/concentration of intratumoral androgens); activation of the AR program via feedback pathways (e.g. AKT/mTOR/Pi3K, HER2/Neu); and activation of the AR program via mutation or substitution (e.g. AR ligand binding domain mutation; AR splice variants; Glucocorticoid Receptor signaling).[5-21] One of the more well described resistance mechanisms is the emergence of alternatively spliced AR variants, which maintain constitutive activity in spite of lacking the AR ligand-binding domain. Indeed, one AR-V, the AR splice variant 7 (AR-V7), has recently been shown in a prospective study to correlate with a lack of response to Abi and Enza.[5] The emergence of AR-Vs provides an elegant biologic rationale for why drugs that interfere with the AR-ligand interaction may not

be effective in patients harboring AR-Vs at significant levels.[16,22-26] An agent that can effectively disrupt one or more of these resistance mechanisms would be an invaluable therapeutic option for men who develop resistance to Abi or Enza.

Niclosamide, an antihelminthic drug used in the treatment of tapeworms, has been used in humans for several decades and is generally well tolerated.[27] Recently, a drug library screen of over 1,100 FDA approved agents identified niclosamide as being a potent AR-V7 inhibitor, suppressing AR-V7 transcripts and protein expression.[28] Importantly, niclosamide has also been shown to exert an anti-neoplastic effect in both *in vitro* and *in vivo* AR-V7 prostate cancer models, with *in vitro* studies demonstrating cell line growth inhibition when these cell lines are exposed to niclosamide concentrations as low as 0.25 µg/mL for 48 hours. A synergistic anti-tumor effect is also observed when niclosamide is combined with enzalutamide or abiraterone.[28,29] Interestingly, in addition to its effect on AR-V7, niclosamide has been found to inhibit multiple other pathways implicated in prostate cancer proliferation, resistance and oncogenesis.[30] Other than the AR-signaling pathway, those pathways inhibited by niclosamide that represent viable therapeutic targets for men with prostate cancer include: NF-κB, Wnt/β-catenin and mTOR signaling pathways.[31-42] Given that niclosamide may exert an anti-tumor effect through a broad range of mechanism provides justification to explore its use in men with mCRPC.

Niclosamide does have a major limitation in that its oral bioavailability is quite variable, with maximal serum concentrations (C_{max}) following a single 2 gm oral dose ranging from 0.25 to 6.0 µg/mL.[27] Fortunately, the lower bound of this C_{max} range still falls within the range of concentrations previously shown to exert an anti-neoplastic effect on prostate cancer cells, indicating that oral niclosamide may be a viable treatment option for men with mCRPC. However, given that pre-clinical experiments typically exposed cell lines to a continual concentration of niclosamide above 0.25 µg/mL, dosing strategies (i.e. higher dosages; more frequent dosing schedules) aimed at maintaining stable serum drug levels above this threshold concentration of niclosamide (i.e. ≥0.25 µg/mL) should be explored.

Treatment Plan: This is an open label, single-institution, Phase I dose-escalation study designed to determine the safety of TID niclosamide dosing when given in combination with enzalutamide. Our goal is to develop a niclosamide dosing strategy that will safely result in high, consistent serum drug levels. In order to accomplish this, we will administer niclosamide more frequently and at higher doses than those used to treat helminth infections. The primary objective is to determine the safety and tolerability of oral (PO) TID niclosamide combined with

enzalutamide in men with castration-resistant prostate cancer (CRPC) that has progressed on abiraterone. As secondary objectives, we will also assess the pharmacokinetic (PK) profile of TID niclosamide; its effect on PSA; its effect on androgen receptor splice variant (AR-V) mRNA expression; and its effect on protein expression and the transcription program of circulating tumor cells (CTCs).

Eligible patients must have CRPC, defined as progressive prostate cancer (per PCWG2 or RECIST criteria) in spite of a castrate serum testosterone level (i.e. testosterone <50 ng/dL); and have progressed on Abi and plan to move on to Enza therapy.[43] All patients will receive niclosamide at one of the following dose levels: Cohort 1) 500 mg PO TID; Cohort 2) 1000 mg PO TID; and Cohort 3) 1500 mg PO TID. Niclosamide will be tested in combination with enzalutamide since preclinical experiments have demonstrated synergy between these agents, possibly due to the ability of enzalutamide to inhibit full length AR, which is largely unaffected by niclosamide (see Study Schema).[28] Serum niclosamide levels will be determined using LC/MS, and blood samples used to determine pharmacokinetic parameters will be drawn at the following time points after the first dose: 0.5 hr, 1 hr, 1.5 hr, 2 hr, 3 hr, 4 hr, 6 hr, 8 hr and 15 days. Pharmacokinetic parameters of interest include: C_{max}, C_{min}, C_{ss} and $t_{1/2}$.

Given that we anticipate a low rate of toxicity with niclosamide, dose escalation between cohorts will occur according to the continual reassessment method (CRM) so as to maximize the number of patients treated at the highest-dose level.[44] In the event that DLTs occur more frequently than anticipated at the lowest dose cohort (i.e. Niclosamide 500 mg PO TID), the niclosamide dose will de-escalate to 500 mg PO BID. If DLTs occur more frequently than anticipated at the 500 mg PO BID level, the study will terminate prematurely. Niclosamide will be given for a total of 28-days, after which patients will come off study. At that point, treating physicians will be encouraged to continue standard of care enzalutamide if deemed appropriate.

Study Schema:

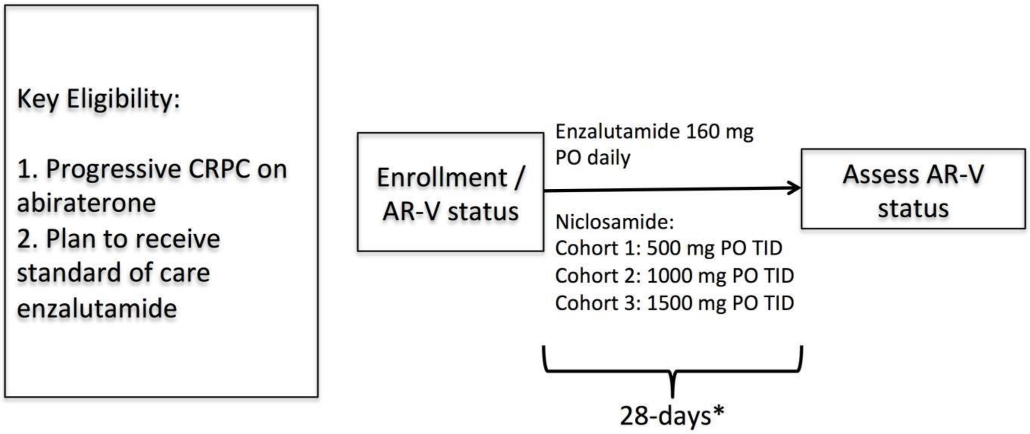

*Safety will be continually assessed during the 28-day niclosamide treatment period. Pharmacokinetic assessments will occur at multiple time points on Day 1 and once on Day 15.

Number of Patients: Up to 12 men with castration-resistant prostate cancer.

Inclusion Criteria:

1. Have signed an informed consent document indicating that the subject understands the purpose of and procedures required for the study and are willing to participate in the study
2. Be willing/able to adhere to the prohibitions and restrictions specified in this protocol
3. Male aged 18 years and above
4. Eastern cooperative group (ECOG) performance status ≤2
5. Documented histologically confirmed adenocarcinoma of the prostate
6. Patient must have evidence of castration resistant prostate cancer as evidenced by a confirmed rising PSA (per Prostate Cancer Working Group 2 [PCWG2] criteria) and a castrate serum testosterone level (i.e. ≤ 50 mg/dL)[43]
7. Patient must be eligible for treatment with enzalutamide
8. Patient must have previously progressed on abiraterone (either by PCWG2 criteria or RECIST criteria)[43,45]
9. Documented metastatic disease on bone scan, CT scan or MRI.

Exclusion Criteria:

1. Have known allergies, hypersensitivity, or intolerance to enzalutamide or niclosamide or their excipients
2. Ongoing systemic therapy (other than a GnRH agonist/antagonist) for prostate cancer including, but not limited to:
    a. CYP-17 inhibitors (e.g. ketoconazole, abiraterone)
    b. Antiandrogens (e.g. bicalutamide, nilutamide)
    c. Second generation antiandrogens (e.g. ARN-509)
    Note: patients receiving ongoing treatment with enzalutamide will be allowed to join the study
    d. Immunotherapy (e.g. sipuleucel-T, ipilimumab)
    e. Chemotherapy (e.g. docetaxel, cabazitaxel)
    f. Radiopharmaceutical therapy (e.g. radium-223, strontium-89, samarium-153)
3. Have any condition that, in the opinion of the investigator, would compromise the well-being of the subject or the study or prevent the subject from meeting or performing study

requirements

4. Any psychological, familial, sociological, or geographical condition that could potentially interfere with compliance with the study protocol and follow-up schedule.
5. Severe hepatic impairment (Child-Pugh Class C)
6. Severe renal impairment (creatinine clearance ≤30 ml/min)
7. History of prior seizures
8. Central nervous system metastases
9. Symptomatic patients who, in the opinion of the investigator, may benefit from docetaxel-based chemotherapy

Primary Objective: Determine the safety and tolerability of three-times-daily (TID) oral niclosamide combined with enzalutamide in men with castration-resistant prostate cancer (CRPC) that has progressed on abiraterone.

Secondary Objectives:

1. Determine the effect of niclosamide plus enzalutamide on androgen receptor splice variant (AR-V) expression as determined by qRT-PCR.
2. Determine the pharmacokinetic profile of three-times-daily (TID) oral niclosamide in men with castration-resistant prostate cancer (CRPC) that has progressed on abiraterone.
3. Determine the PSA response rate (i.e. proportion of subjects with ≥50% decline in PSA from pre-study baseline) after 28-days of niclosamide plus enzalutamide.
4. Determine the effect of niclosamide plus enzalutamide on protein expression and the transcriptional program of circulating tumor cells.

Statistical Considerations: Anticipating low toxicity for niclosamide, we propose an alternative to the standard 3+3 dose escalation trial design; we will use the continual reassessment method (CRM) to evaluate dose-related toxicities and to determine the recommended phase 2 dose.[44] If the expected low toxicity holds, relative to a 3+3 design, the CRM will escalate to higher dose levels faster, treat more patients at higher dose levels, and provide greater power for determining whether the steady-state serum concentration exceeds levels shown to inhibit AR-V7 expression in prostate cancer cell lines. If dose-limiting toxicities accumulate faster than anticipated, dose levels will de-escalate accordingly.[46] Adverse events will be documented by incidence and severity graded according to the National Cancer Institute – Common Terminology Criteria for Adverse Events (CTCAE) version 4.0.

We will target a maximum 30% of patients incurring dose-limiting toxicities up to 30 days after the final (day 28) dose. Because we anticipate low toxicity, however, we do not expect to achieve this maximum toxicity, and consequently we pre-specify stopping the trial after accruing a maximum of 12 patients. For comparison with the 3+3 design, we use a 1-parameter power model for the dose-toxicity relationship with a Gamma(1,1) prior distribution. The table below shows 3 dose-toxicity scenarios and corresponding frequencies of patients who receive each dose level under the CRM and 3+3 designs.[47] The CRM consistently assigns a higher frequency of patients to the highest dose. Note that in all scenarios the risk of dose-limiting toxicities at the highest dose does not exceed the maximum toxicity level of 30%.

Dose-toxicity Scenarios: Prior guesses of risks of dose-limiting toxicities and comparison of frequencies of patients receiving each dose level in CRM and 3+3 designs under 3 scenarios.

	**Dose level (PO TID)**		
	500 mg	1000 mg	1500 mg
**Scenario 1**			
Actual risk	5%	5%	5%
CRM (3+3) frequency	27% (35%)	25% (33%)	48% (32%)
**Scenario 2**			
Actual risk	5%	10%	15%
CRM (3+3) frequency	27% (34%)	26% (35%)	48% (31%)
**Scenario 3**			
Actual risk	5%	20%	30%
CRM (3+3) frequency	28% (38%)	31% (39%)	41% (23%)

To examine statistical power for the secondary PK/PD endpoint, motivated by Andrews et al., we assume niclosamide steady-state concentration follows a normal distribution with mean 2.9 and standard deviation 1.4 μg/mL.[27] Allowing a 5% probability of a type 1 error, N=12 patients yields over 99% power to conclude that the mean steady-state concentration exceeds 0.25 μg/mL based on a 1-sided 1-sample t-test. If the mean steady-state concentration is lower, the variance is greater, or the distribution is positively skewed, power will be smaller. Under a wide range of possibilities, however, N=12 patients will provide sufficient power to test whether the mean steady-state concentration exceeds this target.

Mean niclosamide concentration *vs.* time will be plotted for each dose cohort. Pharmacokinetic parameters (i.e. C_{max}, C_{min}, C_{ss} and $t_{1/2}$) will be reported as means for each

dose cohort along with the observed ranges. The percent change in PSA following 28-days of combination therapy will be presented as a waterfall plot, with the rate of PSA response (i.e. ≥50% decline in PSA from baseline) reported as percentages with 95% confidence intervals.

SYNOPSIS.. 2

Study schema... 4

1. INTRODUCTION 11

1.1 Overview and Rationale.. 11

1.2 AR-Directed Therapies in Prostate Cancer 12

1.3 Niclosamide Background 17

1.4 Overcoming Resistance with Niclosamide 17

1.5 Rationale for Dosage Selection.. 20

2. STUDY OBJECTIVES 22

2.1 Primary Objective... 22

2.2 Secondary Objectives.. 22

3. PATIENT POPULATION AND SELECTION 23

3.1 Inclusion Criteria.. 23

3.2 Exclusion Criteria 23

3.3 Inclusion of Minorities and Women 24

4. TREATMENT PLAN.. 25

4.1 Study Design 25

4.2 Study Schema 26

4.3 Dose Limiting Toxicities... 26

4.4 Removal of Patients from Study.. 27

5. Treatment Assessments..29

5.1 Screening Studies.. 29

5.2 Treatment Phase 29

5.3 Off Study Visit 30

5.4 Follow-up Visit 30

6. STUDY CALENDAR 31

7. STUDY ASSESSMENTS 32

7.1 AR Splice Variant qRT-PCR... 32

7.2 Niclosamide Pharmacokinetics... 32

7.3 Circulating Tumor Cell Profiling……. 32

7.4 PSA Response……. 32

7.5 Safety Assessments 32

8. PHARMACEUTICAL INFORMATION 33

8.1 Niclosamide…….. 33

8.2 Enzalutamide …. 35
9. DATA MONITORING AND REPORTING REQUIREMENTS 41
9.1 Lead Site Principal Investigator 41
9.2 Definitions 41
9.3 Evaluating Adverse Events 43
9.4 Documenting Adverse Events 44
9.5 Adverse Event Monitoring and Reporting 45
9.6 Withdrawal from Study 47
10. STATISTICAL METHODS 48
11. REFERENCES 50

1. Introduction

1.1. Overview and Rationale

It is estimated that over 230,000 American men will be diagnosed with prostate cancer in 2014, with nearly 30,000 affected individuals expected to die as a result of their disease.[48] Androgen ablation therapy has remained the standard of care for men with advanced prostate cancer since its discovery by Charles Huggins in the 1940s.[49] While androgen ablation provides significant palliative benefit, its therapeutic benefits are not indefinite and the majority of these men eventually progress to a state known as metastatic castration-resistant prostate cancer (mCRPC).[43] The observation that androgen receptor (AR) regulated genes (e.g. PSA) remain expressed in a castrate state led to the further exploration of the AR-signaling axis as a therapeutic target in men with mCRPC. These findings have led to the development of effective new AR-directed agents like abiraterone and enzalutamide which inhibit AR-signaling in men with mCRPC through disrupting the ligand-AR interaction (abiraterone through ligand depletion and enzalutamide through receptor antagonism).[1-4] These agents are unfortunately not curative, however, with resistance typically occurring within one year.

A number of mechanisms have been described by which resistance occurs to these next-generation AR-directed agents, including: alterations leading to persistent canonical AR-signaling (e.g. AR amplification/overexpression, elucidations/concentration of intratumoral androgens); activation of the AR program via feedback pathways (e.g. AKT/mTOR/Pi3K, HER2/Neu); and activation of the AR program via mutation or substitution (e.g. AR ligand binding domain mutation; AR splice variants; Glucocorticoid Receptor signaling).[5-21] One of the more well described resistance mechanisms is the emergence of alternatively spliced AR variants, which maintain constitutive activity in spite of lacking the AR ligand-binding domain.[16,26] Indeed, one AR-V, the AR splice variant 7 (AR-V7), has recently been shown in a prospective study to correlate with a lack of response to abiraterone and enzalutamide.[5] The emergence of AR-Vs provides an elegant biologic rationale for why, in some cases, drugs that interfere with the AR-ligand interaction may not be effective in patients harboring AR-Vs at significant levels.[16,22-26] However, AR-Vs likely only account for ~50% of cases of resistance in patients previously treated with abiraterone or enzalutamide.[5] Other pathways implicated in promoting prostate cancer resistance include: NF-κB, Wnt/β-catenin and mTOR signaling pathways.[31-39] An agent that can

effectively disrupt one or more of these resistance mechanisms would be an invaluable therapeutic option for men who develop resistance to abiraterone or enzalutamide.

Niclosamide, an FDA approved anti-helminthic, has recently been shown to be active in AR-V positive prostate cancer models as well as to inhibit other pathways implicated in prostate cancer resistance and progression.[28,31,40-42] Given the need to develop effective drugs for men with the most resistant forms of mCRPC, and compelling preclinical evidence for its broad reaching anti-tumor properties, work towards developing niclosamide as a repurposed prostate cancer therapy should be pursued.

1.2. Androgen Receptor Directed Therapies in Prostate Cancer

The initial management of advanced prostate cancer involves targeting the androgen/androgen receptor (AR) signaling axis through lowering serum testosterone levels to the castrate range (i.e. testosterone <20-50 ng/dL); accomplished either through surgical castration or now more commonly medical castration with a GnRH agonist/antagonist [i.e., hormonal therapy (HT)].[50,51] Short-term anti-androgen therapy (e.g. bicalutamide, nilutamide) as well as docetaxel-based chemotherapy also play a role in managing men with newly diagnosed metastatic disease.[52] This treatment approach is initially highly effective, leading to objective responses in >85% of individuals; however, after a variable period of symptom relief, androgen ablation invariably ceases to suppress prostate cancer growth and patients eventually succumb to their disease.[53] This disease state defined by progression in spite of androgen ablation has been termed metastatic castrate resistant prostate cancer (mCRPC) (Figure 1).

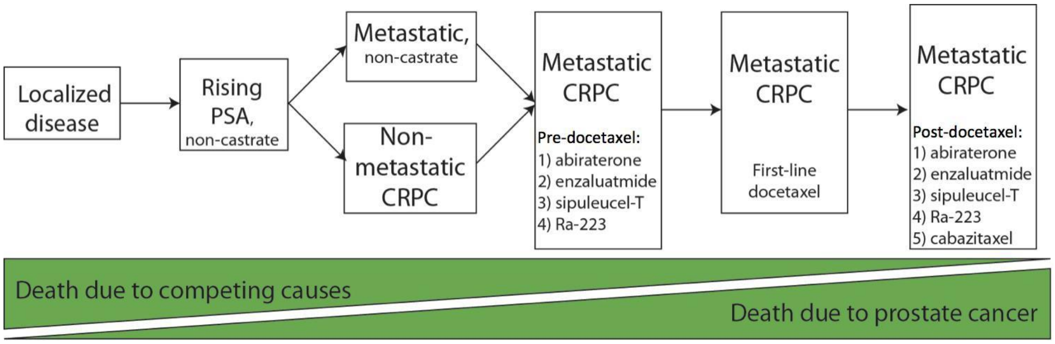

Figure 1: Prostate cancer clinical states model (adapted from Scher et al, 2000).[54]

Recently it has come to light that mCRPC is still largely dependent on androgen/AR axis signaling.[55] This realization has led to the development of multiple newer AR directed therapies. Mechanistically these agents primarily work either through ligand depletion (e.g., abiraterone) or through interference with AR trafficking and signaling (e.g. enzalutamide).[50] The current standard treatment of mCRPC entails the continuation of hormonal therapy with the sequential administration of a number of secondary androgen-directed agents (e.g. abiraterone, enzalutamide), immunotherapeutics (e.g. sipuleucel-t), radiopharmaceuticals (e.g. radium-223) and/or chemotherapeutics (e.g. docetaxel, cabazitaxel) (Figure 1).[1-4,50,56-58]

1.2.1. Enzalutamide

Enzalutamide works through competitive AR inhibition and, unlike the older anti-androgens (e.g. bicalutamide, nilutamide), is a pure AR antagonist. It additionally has the ability to prevent AR nuclear translocation and DNA binding to nuclear response elements.[50] It was approved in 2012 for men with mCRPC post-docetaxel.[4] It is expected to gain approval for men who are docetaxel-naïve given that a recently completed Phase III study showed OS improvements in a pre-chemotherapy population as well.[1] In that patient population enzalutamide was shown to result in a 37% reduced risk of death compared to placebo (HR 0.63, 95% CI, 0.53-0.75; P<0.001).[4]

1.2.1.1. Enzalutamide Clinical Trial Experience

Enzalutamide was initially tested in a Phase I/II dose escalation trial.[59] In that study enzalutamide was tested at doses ranging from 30 mg to 600 mg by mouth daily. It was found to exert an antitumor effect at all doses tested, with a PSA response rate (i.e. ≥50% PSA declines) in 78 out of 140 enrolled subjects (56%). Fatigue was the most common adverse event (AE) and generally occurred following 30-days of treatment. At doses ≥240 mg daily, an increasing proportion of patients required dose reductions secondary to fatigue. Overall grade 3-4 AEs included: fatigue (11%), anemia (3%), arthralgia (2%), asthenia (2%) and seizures (2%). Overall mild (i.e. grade 2) AEs included: fatigue (27.1%), nausea (8.6%), dyspnea (7.9%), anorexia (5.7%) and back pain (5.7%). Two witnessed seizures and one questionable seizure occurred in patients receiving doses of 600 mg, 360 mg and 480 mg, respectively. Only 1/87 patients treated at a daily dose of ≤240 mg discontinued

treatment for an AE, compared to 7/53 at doses ≥360 mg. While 240 mg daily was deemed the maximum tolerated dose, it was noted that extent and proportion of patients achieving PSA decreases plateaued somewhere between 150 mg and 240 mg daily. As such, enzalutamide 160 mg by mouth daily was ultimately selected for further investigation.

On the basis of the aforementioned Phase I/II results, the AFFIRM trial, a large randomized Phase III study powered to detect differences in survival, was launched.[4] This enrolled men with CRPC who had already progressed on docetaxel. Subjects were randomized 2:1 between enzalutamide 160 mg daily (N=800) and placebo (N=399). This study met its primary endpoint, demonstrating a median OS of 18.4 months with enzalutamide compared to 13.6 months with placebo (HR for death, 0.63; 95% CI, 0.53 to 0.75; P<0.001). The rate of AEs between enzalutamide and placebo were similar. Enzalutamide had a lower rate of grade 3 or higher AEs compared to placebo (45.3% vs 53.1%, respectively). Rates of fatigue, diarrhea and hot flashes were higher with enzalutamide (table 2). There were notably 5 patients (0.6%) that experienced a seizure in the enzalutamide arm compared to zero in the placebo arm. One of these seizure required medical intervention, the others were self-limited. Predisposing factors were present in several patients, and included: two subjects with brain metastases, one subject inadvertently received IV lidocaine prior to the seizure and one subject had brain atrophy and a history of heavy alcohol use. The results from this study ultimately lead to the approval of enzalutamide in men with CRPC who had already received prior docetaxel.

More recently, the results of another randomized Phase III study, the PREVAIL trial, were released.[1] This study randomized men with CRPC who were docetaxel naïve between enzalutamide 160 mg daily (N=871) and placebo (N=844). As was the case with AFFIRM, the PREVAIL trial demonstrated an OS advantage with enzalutamide. The median OS improved from 30.2 months to 32.4 months with enzalutamide treatment (HR for death, 0.706; 95% CI, 0.60 to 0.84; P<0.0001). While improvement in survival is modest, it should be noted that those in the placebo group received other proven prostate cancer therapies at a

higher rate than those on the enzalutamide arm (e.g. 32.8% vs 56.7% received docetaxel and 20.5% vs 45.6% received abiraterone). AEs occurred at comparable rates between the enzalutamide and placebo arms.

	PREVAIL				AFFIRM			
	Enzalutamide (N=871)		Placebo (N=844)		Enzalutamide (N=800)		Placebo (N=399)	
**Adverse Event**	**All Grades (%)**	**Grade 3-4 (%)**	**All Grades (%)**	**Grade 3-4 (%)**	**All Grades (%)**	**Grade 3-4 (%)**	**All Grades (%)**	**Grade 3-4 (%)**
Fatigue	35.6	1.8	25.8	1.9	34	6	29	7
Back pain	27	2.5	22.2	3				
Constipation	22.2	0.5	17.2	0.4				
Diarrhea					21	1	18	0.3
Hot flashes					20	0	10	0
Arthralgias / musculoskeletal pain	20.3	1.4	16	1.1	14	1	10	0.3
Headache					12	0.8	6	0
Cardiac event/disorder	10.1	2.8	7.8	2.1	6.3	1.1	8	2.5
Hypertension	13.4	6.8	4.1	2.3	6.6		3.3	
Abnormal liver function testing	0.9	0.2	0.6	0.1	1	0.4	2	0.8
Seizure	0	0	0.1	0	0.6	0.6	0	0

Table 1: Adverse events observed on the AFFIRM and PREVAIL trials [Scher et al, 2012; Beer et al, 2014].

1.3. Niclosamide Background

Niclosamide is an antihelminthic drug that has been used to treat tapeworm infections in humans for over 50 years.[27,60] It was marketed as Niclocide in the US until voluntarily withdrawn by Bayer Healthcare LLC in July of 1996. The drug is available through compounding pharmacies in the US and is commercially available as 500 mg tables in several other countries. It remains on the World Health Organization's (WHO) List of Essential Medicines.[61] It inhibits oxidative phosphorylation and stimulates ATP activity in mitochondria of cestodes (i.e. tapeworms), killing the scolex and proximal segments of the tapeworm.[60] In adults, a single 2,000 mg oral dose is effective in the treatment of *Taenia saginata* (beef tapeworm), *Diphyllobothrium latum* (fish tapeworm), and *Dipylidium caninum* (dog tapeworm). For *Hymenolipis nana* (dwarf tapeworm) the same dose is used for seven consecutive days. More effective antihelminthic drugs, such as praziquantel, have largely supplanted niclosamide's use in the US.

Niclosamide is generally well tolerated. Its toxicology data was evaluated by the WHO in 1988 and published in "Data Sheet on Pesticides, No. 63, Niclosamide" (WHO/VBC/DS/88.63).[62] Niclosamide was felt to have very low toxicity in mammals (WHO Hazard Class III; slightly hazardous), with a reported oral LD_{50} in rats >5,000 mg/kg body weight. In dogs and cats treated with intraperitoneal or intravenous niclosamide, poisoning resulted in vomiting. In rats treated with niclosamide 5,000 mg/kg/day by mouth for 4-weeks, there was a marginal decrease in hemoglobin concentration. No-effect was seen at a dose level of 2,000 mg/kg/day. Dogs treated with niclosamide showed no toxic effects at doses up to 6,000 mg/day for 4-weeks. In addition, niclosamide did not demonstrate any evidence of carcinogenicity or teratogenicity in rodents. In healthy human volunteers, niclosamide was noted to result in nausea and abdominal pain in approximately 10% of patients following oral dosing. No cases of human niclosamide poisoning/damage have been reported in spite of its use all over the world since 1960.[27] Niclosamide has not been shown to have an effect on hematologic and urinalysis parameters, nor does it influence liver and kidney function tests.[27]

1.4. Overcoming Resistance to AR-Directed Therapies with Niclosamide

The emergence of constitutively active AR splice variants (AR-Vs), AR point mutations and AR-signaling activation via alternative pathways (e.g. AKT/mTOR/Pi3K,

HER kinases, others) have been described and may play a role in the development of resistance to AR-directed therapeutics.[11,20,31-39,63-67] Even with newer drugs like abiraterone or enzalutamide, resistance is still a major clinical problem, and therapeutic strategies aimed at treating men with the most resistant forms of mCRPC are sorely needed. Niclosamide, an FDA approved anti-helminthic, has recently been shown to be active in AR-V positive prostate cancer models as well as to inhibit other pathways implicated in prostate cancer resistance and progression.[28,31,40-42] Given the need to develop effective drugs for heavily pretreated mCRPC patients, and compelling preclinical evidence for its broad reaching anti-tumor properties, work towards developing niclosamide as a repurposed prostate cancer therapy should be pursued.

As reported by Liu and colleagues, a recent drug library screen of over 1,100 FDA approved agents identified niclosamide as being a potent AR-V7 inhibitor.[28] It was shown to inhibit AR-V7 protein expression, while AR-V7 mRNA transcripts were unaffected. This indicates that niclosamide likely function by enhancing the degradation of AR-V7 protein. In addition, treating cells with the 26S proteasome inhibitor MG132 reduced niclosamide mediated inhibition of AR-V7 protein expression, indicating that niclosamide likely functions through enhancing the ubiquitin–proteasome system. Liu, *et al* also demonstrated that niclosamide was able to exert an anti-neoplastic effect in *in vitro* and *in vivo* AR-V7 prostate cancer models, with *in vitro* studies demonstrating that niclosamide induced apoptosis and inhibited cell line growth at concentrations as low as 0.25 µg/mL.

A synergistic anti-tumor effect is also observed when niclosamide is combined with enzalutamide or abiraterone (Figure 2).[28,29] Of note, full length AR protein expression appears to be unaffected by niclosamide; therefore, it seems plausible that this synergy is a result of niclosamide selectively targeting AR-V7 expression or another oncogenic signaling pathway. Co-targeting of full length AR will therefore likely be necessary given that canonical AR signaling is still active in patients with CRPC. Based on these observations, therapeutic strategies utilizing niclosamide will almost certainly require concurrent treatment with drugs designed to prevent full length AR-signaling (e.g. abiraterone or enzalutamide).

It should be noted that while there is a strong rationale for targeting AR-V7, there are a number of resistance mechanisms contributing to disease progression following treatment with abiraterone or enzalutamide. Indeed, while initial estimates found that

AR-V7 transcripts were present in ~50% of men progressing on abiraterone or enzalutamide, larger studies have found that only ~25% of men have evidence of an AR-V after progressing on one of these AR-directed agents.[5,68] Other recent papers have reported that niclosamide is able to inhibit NF-κB, Wnt/β-catenin and mTOR signaling pathways, all potentially important to promoting prostate cancer growth and resistance.[28,31,40-42]

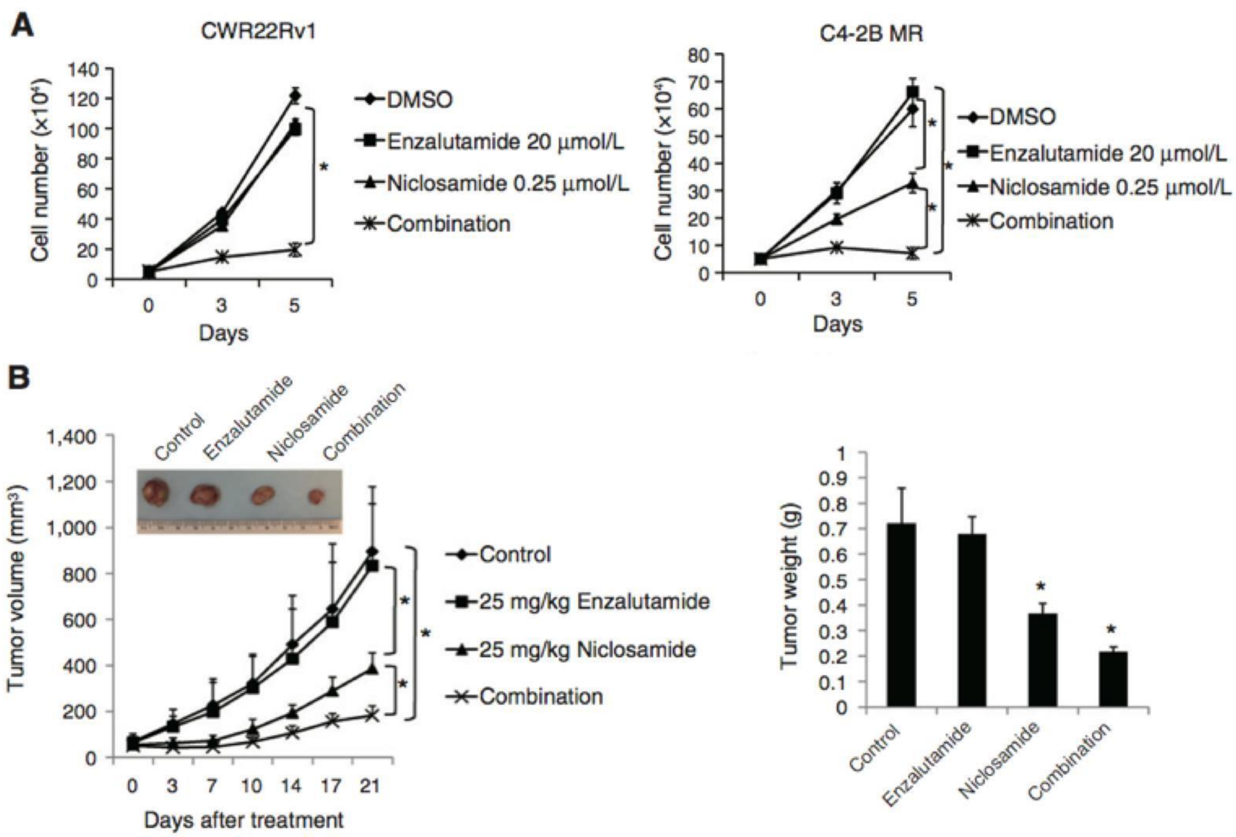

Figure 2: Niclosamide enhances enzalutamide effects (from Liu et al, 2014).[28] A) AR-V7 positive CWR22Rv1 and C4-2B MR cells treated with 0.25 µmol/L niclosamide with or without 20 µmol/L enzalutamide. B) Mice bearing CWR22Rv1 xenografts treated with vehicle control, enzalutamide, or their combination for 3-weeks.

1.4.1. Niclosamide Pharmacokinetics

Once daily dosing of niclosamide is well tolerated, with only occasional gastrointestinal (GI) side effects.[27,69] It is only partially absorbed through the GI

track and the absorbed fraction is rapidly eliminated by the kidneys.[27] Published data regarding the pharmacokinetics of niclosamide is somewhat limited, however. In a cohort of healthy male and female volunteers administered a single 2 gm dose of carbonyl-^{14}C-labeled niclosamide, the fraction of ^{14}C-activity eliminated in the urine was 2-25% over 4 days. The remainder was eliminated in the feces. Elimination was almost complete within 1-2 days. In this study, C_{max} was found to be 0.25 to 6.0 µg/mL (0.76 to 18.35 µM). Fortunately, the lower bound of this C_{max} range still falls within the range of concentrations previously shown to exert an anti-neoplastic effect on resistant prostate cancer cells, indicating that oral niclosamide may be effective in CRPC patients.[28] However, given that cell culture models of CRPC typically exposed cell lines to a continual concentration of niclosamide above 0.25 µg/mL, dosing strategies (i.e. higher dosages; more frequent dosing schedules) aimed at maintaining stable serum drug levels above this threshold concentration of niclosamide (i.e. ≥0.25 µg/mL) should be explored.

1.5. Rationale for Dosage Selection

1.5.1. Enzalutamide

An enzalutamide dose of 160 mg by mouth daily has been tested in men with metastatic CRPC in two separate Phase III studies and is currently FDA approved at this dose/schedule on the basis of data showing that it leads to an OS advantage compared to placebo.[1,4] We will therefore plan to administer enzalutamide at this fixed dose/schedule throughout the study.

1.5.2. Niclosamide

Niclosamide is indicated to treat intestinal tapeworm infections as a single, 2,000 mg oral dose. Given that this dose has been shown to: i) result in relatively low plasma concentrations, and ii) be rapidly excreted; we plan to explore alternative dosing regimens aimed at achieving high, consistent plasma drug levels. In order to accomplish this we will dose niclosamide more frequently and at higher doses. Therefore, three-time-daily (TID) dosing was chosen over daily dosing. Given that niclosamide is typically formulated as 500 mg tablets, we plan to escalate each dose in 500 mg increments as follows: 1) Niclosamide 500

mg PO TID, 2) Niclosamide 1000 mg PO TID, and 3) Niclosamide 1500 mg PO TID.

2. Study Objectives

2.1. Primary Objective

Determine the safety and tolerability of three-times-daily (TID) oral niclosamide combined with enzalutamide in men with castration-resistant prostate cancer (CRPC) that has progressed on abiraterone.

2.2. Secondary Objectives

1. Determine the effect of niclosamide plus enzalutamide on androgen receptor splice variant (AR-V) expression as determined by qRT-PCR.
2. Determine the pharmacokinetic profile of three-times-daily (TID) oral niclosamide in men with castration-resistant prostate cancer (CRPC) that have progressed on abiraterone.
3. Determine the PSA response rate (i.e. proportion of subjects with ≥50% decline in PSA from pre-study baseline) after 28-days of niclosamide plus enzalutamide.
4. Determine the effect of niclosamide plus enzalutamide on protein expression and the transcriptional program of circulating tumor cells.

3. Patient Population and Selection

3.1. Inclusion Criteria:

1. Have signed an informed consent document indicating that the subject understands the purpose of and procedures required for the study and are willing to participate in the study
2. Be willing/able to adhere to the prohibitions and restrictions specified in this protocol
3. Male aged 18 years and above
4. Eastern cooperative group (ECOG) performance status ≤2
5. Documented histologically confirmed adenocarcinoma of the prostate
6. Patient must have evidence of castration resistant prostate cancer as evidenced by a confirmed rising PSA (per Prostate Cancer Working Group 2 [PCWG2] criteria) and a castrate serum testosterone level (i.e. ≤ 50 mg/dL)[43]
7. Patient must be eligible for treatment with enzalutamide
8. Patient must have previously progressed on abiraterone (either by PCWG2 criteria or RECIST criteria)[43,45]
9. Documented metastatic disease on bone scan, CT scan or MRI.

3.2. Exclusion Criteria:

1. Have known allergies, hypersensitivity, or intolerance to enzalutamide or niclosamide or their excipients
2. Ongoing systemic therapy (other than a GnRH agonist/antagonist) for prostate cancer including, but not limited to:
    a. CYP-17 inhibitors (e.g. ketoconazole, abiraterone)
    b. Antiandrogens (e.g. bicalutamide, nilutamide)
    c. Second generation antiandrogens (e.g. ARN-509)
       Note: patients receiving ongoing treatment with enzalutamide will be allowed to join the study
    d. Immunotherapy (e.g. sipuleucel-T, ipilimumab)
    e. Chemotherapy (e.g. docetaxel, cabazitaxel)
    f. Radiopharmaceutical therapy (e.g. radium-223, strontium-89, samarium-153)
3. Have any condition that, in the opinion of the investigator, would compromise the well-being of the subject or the study or prevent the subject from meeting or performing study requirements

4. Any psychological, familial, sociological, or geographical condition that could potentially interfere with compliance with the study protocol and follow-up schedule.
5. Severe hepatic impairment (Child-Pugh Class C)
6. Severe renal impairment (creatinine clearance ≤30 ml/min)
7. History of prior seizures
8. Central nervous system metastases
9. Symptomatic patients who, in the opinion of the investigator, may benefit from docetaxel-based chemotherapy

3.3. Inclusion of Women and Minorities

This study is focused on prostate cancer; therefore the treatment cohort is only applicable to men. Men from all ethnic and race groups are eligible for this study.

4. Treatment Plan

4.1. Study Design

This is an open label, single-institution, Phase I dose-escalation study designed to determine the safety of TID niclosamide dosing when given in combination with enzalutamide. Our goal is to develop a niclosamide dosing strategy that will safely result in high, consistent serum drug levels. In order to accomplish this, we will administer niclosamide more frequently and at higher doses than those used to treat helminth infections. The primary objective is to determine the safety and tolerability of oral (PO) TID niclosamide combined with enzalutamide in men with castration-resistant prostate cancer (CRPC) that has progressed on abiraterone. As secondary objectives, we will also assess the pharmacokinetic (PK) profile of TID niclosamide; its effect on PSA; its effect on androgen receptor splice variant (AR-V) mRNA expression; and its effect on protein expression and the transcription program of circulating tumor cells (CTCs).

Eligible patients must have CRPC, defined as progressive prostate cancer (per PCWG2 or RECIST criteria) in spite of a castrate serum testosterone level (i.e. testosterone <50 ng/dL); and have progressed on Abi and plan to move on to Enza therapy;.[43] All patients will receive niclosamide at one of the following dose levels: Cohort 1) 500 mg PO TID; Cohort 2) 1000 mg PO TID; and Cohort 3) 1500 mg PO TID. Niclosamide will be tested in combination with enzalutamide since preclinical experiments have demonstrated synergy between these agents, possibly due to the ability of enzalutamide to inhibit full length AR, which is largely unaffected by niclosamide (see Study Schema).[28] Serum niclosamide levels will be determined using LC/MS, and blood samples used to determine pharmacokinetic parameters will be drawn at the following time points after the first dose: 0.5 hr, 1 hr, 1.5 hr, 2 hr, 3 hr, 4 hr, 6 hr, 8 hr and 15 days. Pharmacokinetic parameters of interest include: C_{max}, C_{min}, C_{ss} and $t_{1/2}$.

Given that we anticipate a low rate of toxicity with niclosamide, dose escalation between cohorts will occur according to the continual reassessment method (CRM) so as to maximize the number of patients treated at the highest-dose level.[44] In the event that DLTs occur more frequently than anticipated at the lowest dose cohort (i.e. Niclosamide 500 mg PO TID), the niclosamide dose will de-escalate to 500 mg PO BID. If DLTs occur more frequently than anticipated at the 500 mg PO BID level, the study will terminate prematurely. Niclosamide will be given for a total of 28-days, after which patients will come off study. At

that point, treating physicians will be encouraged to continue standard of care enzalutamide if deemed appropriate.

4.2. Study Schema

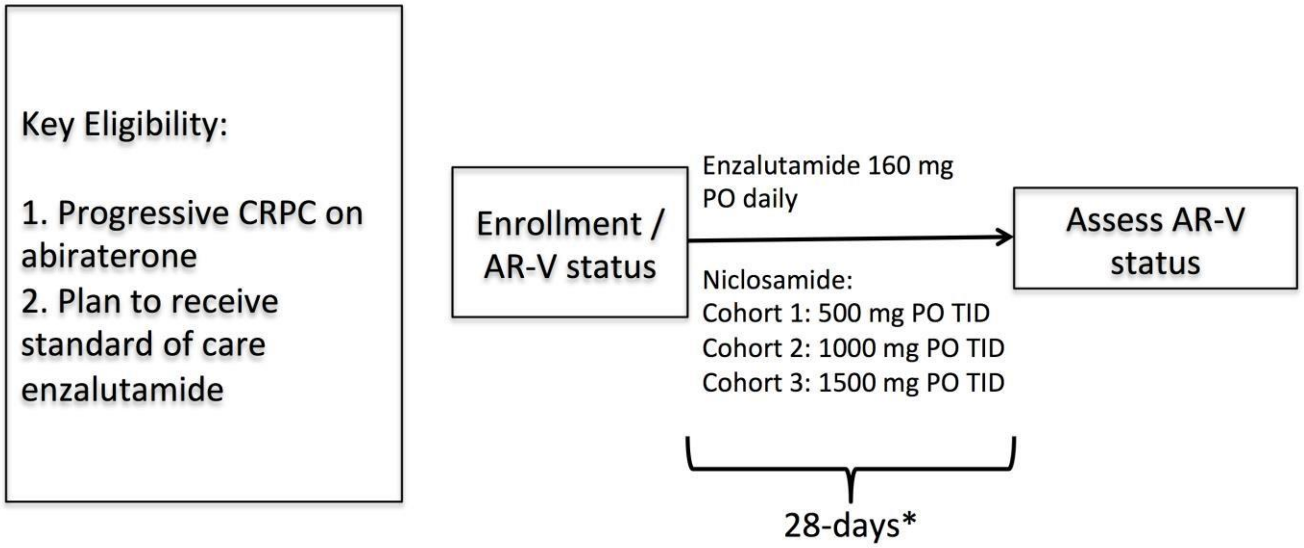

Figure 3: Treatment Schema.

*Safety will be continually assessed during the 28-day niclosamide treatment period. Pharmacokinetic assessments will occur at multiple time points on Day 1 and once on Day 15.

4.3. Dose Limiting Toxicities

Prior experience with niclosamide indicates that it is very well tolerated, with minimal associated toxicities.[27,60] The most commonly observed side effects associated with niclosamide use are gastrointestinal in nature (~10% of patients), including nausea, vomiting and stomach pain. At high doses, rodent studies have indicated that niclosamide can result in marginal declines in hemoglobin concentration. Potential dose limiting toxicities (DLTs) include:

- Grade 3-4 diarrhea (CTC AE v4.0: increase of ≥7 stools per day over baseline; incontinence; hospitalization indicated; severe increase in ostomy output compared to baseline; limiting self care ADL) in spite of optimal use of anti-diarrheal medications
- Grade 4 vomiting (CTC AE v4.0: life-threatening consequences; urgent intervention indicated)

- Grade 3 vomiting lasting >72 hours (CTC AE v4.0: ≥6 episodes [separated by 5 minutes] in 24 hours; tube feeding, TPN or hospitalization indicated)
- Grade 3 nausea lasting >72 hours (CTC AE v4.0: Inadequate oral caloric or fluid intake; tube feeding, TPN, or hospitalization indicated)
- Grade 3-4 abdominal pain (CTC AR v4.0: severe pain; limiting self care ADL)
- Grade 4 anemia (CTC AR v4.0: life-threatening consequences; urgent intervention indicated)
- Grade 3-4 non-hematological toxicity except:
    - Grade 3 nausea, vomiting, and diarrhea lasting < 72 hours in patients who have not received maximal medical care
    - Grade 4 vomiting and diarrhea lasting < 72 hours in patients who have not received maximal medical care
- Grade 4 neutropenia > 5 days (CTC AR v4.0: absolute neutrophil count <500/mm^3)
- Grade 3-4 febrile neutropenia (CTC AR v4.0: absolute neutrophil count <1000/mm^3 with a single temperature of >38.3°C or a sustained temperature of ≥38°C for more than one hour)
- Grade 3 thrombocytopenia with hemorrhage (CTC AR V4.0: platelet count <50,000 – 25,000 mm^3)
- Grade 4 thrombocytopenia (CTC AR V4.0: platelet count <25,000 mm^3)

4.4. Removal of Patients from Study

A patient may be removed from the study for a variety of reasons, including:

1. Worsening symptoms that can be attributed to prostate cancer
2. Unacceptable adverse event(s)
    - Patients develop urinary outlet obstruction requiring urinary catheterization and/or surgical intervention
    - Patients who develop grade 3 or higher liver function abnormalities:
        - Bilirubin ≥ 3 times institutional upper limit of normal (ULN)
        - AST (SGOT) or ALT (SGPT) ≥ 5 times ULN
    - Patients develop decreased renal function with serum creatinine ≥ 2.5 times baseline level

- Patients develop hypersensitivity or anaphylactoid reactions to enzalutamide or niclosamide.

3. Dose limiting toxicity during the 28-day treatment period
4. Intercurrent illness that prevents further participation
5. Experiencing a treatment delay of longer than 2 weeks due to drug toxicity
6. Patient refuses further treatment through the study and/or withdraws consent to participate
7. Patients is noncompliant with respect to taking drugs, keeping appointments, or having tests required for the evaluation of drug safety and efficacy
8. General or specific changes in the patient's condition that render the patient unacceptable for further treatment in this study in the judgment of the investigator.
9. Under no circumstance will care of a withdrawn patient be adversely affected by a decision to withdraw or be withdrawn from the study.

5. Treatment Assessments

5.1. Screening Studies (Day -30 to -1)

Assessments may be performed any time during this 30-day window.

1. Comprehensive medical history and physical exam, including height and weight, medications, blood pressure and heart rate
2. PSA (Prostate-specific antigen)
3. CBC (Complete blood count) with differential and platelet count
4. CMP (Comprehensive Metabolic Panel - Sodium, Potassium, Chloride, BUN, Serum Creatinine, Calcium, Total Protein, Albumin, Total Bilirubin, AST, ALT, Alkaline Phosphatase, CO_2)
5. Serum testosterone
6. Electrocardiogram (EKG)

5.2. Treatment Phase (Day 1 to 28)

Assessments must be done within ±3 days of the specified time point unless indicated otherwise. All pharmacokinetic studies must be done at the precise time indicated.

1. Comprehensive medical history and physical exam, including height and weight, medications, blood pressure and heart rate (Day 1:Pre-treatment [within the preceding 7 days], Day 8, Day 15)
2. PSA (Day 1:Pre-treatment [within the preceding 7 days])
3. CBC (Complete blood count) with differential and platelet count (Day 1:Pre-treatment [within the preceding 7 days], Day 8, Day 15)
4. CMP (Comprehensive Metabolic Panel - Sodium, Potassium, Chloride, BUN, Serum Creatinine, Calcium, Total Protein, Albumin, Total Bilirubin, AST, ALT, Alkaline Phosphatase, CO_2) (Day 1:Pre-treatment [within the preceding 7 days], Day 8, Day 15)
5. AR-V (Androgen receptor splice variant) qRT-PCR (Day 1: Pre-treatment)
6. CTC (circulating tumor cell) protein expression and transcriptional profiling studies (Day 1: Pre-treatment)

7. Niclosamide PK (pharmacokinetics) (Day 1: 0.5 hours, 1 hour, 1.5 hours, 2 hours, 3 hours, 4 hours, 6 hours, 8 hours; Day 15 [±1 day])

5.3. Off Study Visit (Day 29)

Assessments must be done within ±3 days.

1. Comprehensive medical history and physical exam, including height and weight, medications, blood pressure and heart rate
2. PSA
3. CBC (Complete blood count) with differential and platelet count
4. CMP (Comprehensive Metabolic Panel - Sodium, Potassium, Chloride, BUN, Serum Creatinine, Calcium, Total Protein, Albumin, Total Bilirubin, AST, ALT, Alkaline Phosphatase, CO2)
5. AR-V qRT-PCR
6. CTC (circulating tumor cell) protein expression and transcriptional profiling studies

5.4. Follow Up Visit (Day 58 and 88)

Assessments must be done within ±7 days.

1. Telephone, email or postal follow up
2. PSA
3. CBC (Complete blood count) with differential and platelet count
4. CMP (Comprehensive Metabolic Panel - Sodium, Potassium, Chloride, BUN, Serum Creatinine, Calcium, Total Protein, Albumin, Total Bilirubin, AST, ALT, Alkaline Phosphatase, CO2)

6. Study Calendar

	Screening	Treatment Phase											Off Study Visit	Follow Up Visit	
	Day -30 to -1	Day 1									Day 8	Day 15	Day 29	Day 58 (+/- 7 days)	Day 88 (+/- 7 days)
		Pre-treatment	0.5 hours[b]	1 hours[b]	1.5 hour[b]	2 hour[b]	3 hour[b]	4 hour[b]	6 hour[b]	8 hour[b]					
Informed Consent	x														
History and Physical	x	x[a]									x	x	x		
Niclosamide dispensation		x													
Enzalutamide prescription	x														
Initiate niclosamide plus enzalutamide		x													
EKG	x														
CBC	x	x[a]							x		x	x	x	x	x
CMP	x	x[a]									x	x	x	x	x
Serum PSA	x	x[a]											x	x	x
Serum testosterone	x														
Blood for niclosamide PK			x	x	x	x	x	x	x	x		x			
Blood for AR-V qRT-PCR		x											x		
Blood for CTC protein/transcriptional profiling		x											x		
Telephone, email or postal follow up[c]														x	x

CBC = complete blood count with differential and platelets; CMP = Comprehensive Metabolic Panel (Sodium, Potassium, Chloride, BUN, Serum Creatinine, Calcium, Total Protein, Albumin, Total Bilirubin, AST, ALT, Alkaline Phosphatase, CO2); EKG = Electrocardioram; PK = Pharmacokinetics; AR-V = Androgen Receptor Splice Variant; qRT-PCR = quantitative reverse-transcriptase–polymerase-chain-reaction; CTC = circulating tumor cell.

a. The Screening visit History and Physical, CBC, CMP and PSA may replace the Pre-treatment visit History and Physical, CBC, CMP and PSA if done within the past 7 days.

b. All times are from the first dose of niclosamide

c. Will capture adverse events that have occurred since last time the patient was seen and current medication list.

7. Study Assessments

7.1. AR Splice Variant (AR-V) qRT-PCR

The presence of AR-V transcripts will be determined in Dr. Stephen Plymate's lab from circulating tumor cells (CTC) using qRT-PCR and primers designed to detect AR-V7 and AR-V567e (i.e. exon 5, 6 and 7 deleted AR-V) mRNA. The AlereTM CTC AdnaTest platform (AdnaGen, Langenhagen, Germany) and methods similar to those described by Antonarakis and colleagues will be used.[5] AR-V positivity will be defined as the number of PCR cycles (or fewer) required to detect at least one copy of AR-V complementary DNA based on serial dilution of prequantified AR-V.[5]

7.2. Niclosamide Pharmacokinetics

Niclosamide serum drug levels will determined using liquid chromatography-mass spectrometry (LC-MS). Niclosamide LC-MS assays will be conducted similar to the methods previously described by Bussy and colleagues, and done under the supervision of Dr. Jeannine McCune (University of Washington/Fred Hutchinson Cancer Research Center).[70]

7.3. Circulating Tumor Cell Profiling

In addition to isolating CTCs using the AlereTM CTC AdnaTest Platform as outlined above, we will also capture CTCs for additional transcriptional profiling studies (e.g. RNA-seq, qRT-PCR) and protein expression studies using the RareCyte platform (Seattle, WA). RareCyte is a density-based CTC enrichment platform that utilizes automated immunofluorescence staining and digital microscopy. This work will be exploratory in nature.

7.4. PSA Response

Per the Prostate Cancer Working Group 2 criteria, a PSA response will be defined as a ≥50% decline in PSA compared to baseline (i.e. Day 1: Pre-treatment PSA).[43]

7.5. Safety Assessments

Safety will evaluated based on the incidence, severity, duration, causality, seriousness, and type of adverse events (AEs), and changes in the patient's physical examination, vital signs, and clinical laboratory results. Investigators will use the NCI CTC version 4.0 (published 14 June 2010) to assess the severity of AEs and toxicities.

8. Pharmaceutical Information

8.1. Drug Name: Niclosamide[27,71]

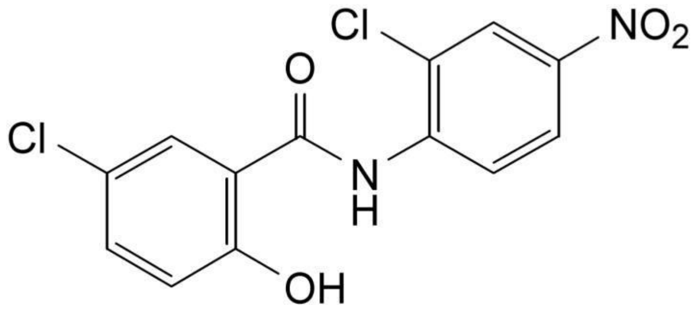

- **Chemical Name:** 2',5-dichloro-4'-nitrosalicylanilide or 5-chloro-N-2-chloro-4-nitrophenyl-2-hydroxybenzamide (top structure).
- **Molecular Formula:** $C_{13}H_8Cl_2N_2O_4$ **Molecular Weight:** 327.11962 g/mol
- **Description:** Niclosamide is a cream-colored or yellowish-white powder. It is almost insoluble in water; sparingly soluble in ethanol, chloroform, or ether; and soluble in acetone. Niclosamide is provided as powder filled gelatin capsules for oral administration. Each capsule contains 500 mg of niclosamide.

8.1.1. Clinical Pharmacology

Once daily dosing of niclosamide is well tolerated, with only occasional gastrointestinal (GI) side effects.[27,69] It is only partially absorbed through the GI track and the absorbed fraction is rapidly eliminated by the kidneys.[27] Published data regarding the pharmacokinetics of niclosamide is somewhat limited, however. In a cohort of healthy male and female volunteers administered a single 2 gm dose of carbonyl-^{14}C-labeled niclosamide, the fraction of ^{14}C-activity eliminated in the urine was 2-25% over 4 days. The remainder was eliminated in the feces. Elimination was almost complete within 1-2 days. In this study, C_{max} was found to be 0.25 to 6.0 µg/mL (0.76 to 18.35 µM).

8.1.2. Safety and Precautions

- **Unforeseeable risks to embryo or fetus:** While rat studies have not demonstrated a risk of embryo or fetal toxicity, patients should be informed that it is not known whether niclosamide or its metabolites are present in semen and they should use a condom if

having sex with a pregnant woman. The patient should use a condom and another effective method of birth control if he is having sex with a woman of child-bearing potential. These measures are required during treatment with niclosamide until one-week after treatment has stopped.

8.1.3. Information for Patients

- Instruct patients to take their doses at the same time each day (three-times-daily). Niclosamide can be taken with or without food.
- Inform patients that niclosamide may cause GI side effects, such as: abdominal discomfort, nausea and vomiting.
- Inform patients that if they miss a dose, then they should take it as soon as they remember. They should not take more niclosamide than indicated by the protocol.
- Apprise patients of the common side effects associated with niclosamide.

8.1.4. Laboratory Tests

No special monitoring needed.

8.1.5. Drug Interactions

No major known drug interactions.

8.1.6. Adverse Reactions

Signs and symptoms associated with niclosamide therapy, though infrequent, include nausea and vomiting, abdominal discomfort including anorexia, diarrhea, drowsiness, and dizziness; those of lesser frequency include constipation, headache, irritability, rash including pruritus ani, alopecia, oral irritation, fever, rectal bleeding, bad taste in mouth, sweating, palpitations, edema of an arm, and backache.

8.1.7. Administration, Supply and Storage

Niclosamide is not approved for the indication under study.

8.1.7.1. Administration

Niclosamide 500 mg, 1,000 mg or 1,500 mg three-times-daily (i.e. one, two or three 500 mg capsules), will be administered.

8.1.7.2. Supply

Niclosamide will be provided in 500 mg soft gelatin capsules filled with a cream-colored or yellowish-white powder.

8.1.7.3. Storage

Niclosamide should be stored at a temperature of <30°C (<86°F). Freezing should be avoided.

8.2. Drug Name: Enzalutamide [Adapted from FDA prescribing information]

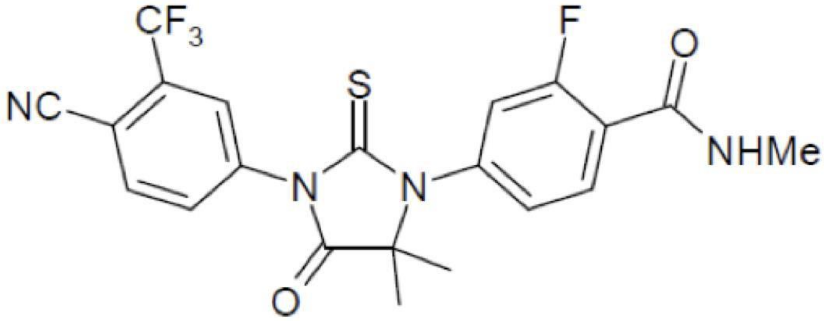

- **Chemical Name:** 4-(3-[4-cyano-3-(trifluoromethyl)phenyl]-5,5dimethyl-4-oxo-2-sulfanylideneimidazolidin-1-yl)-2-fluoro-*N*-methylbenzamide
- **Molecular Formula:** $C_{21}H_{16}F_4N_4O_2S$ **Molecular Weight:** 464.44 g/mol
- **Description:** Enzalutamide is a white crystalline non-hygroscopic solid. It is practically insoluble in water. Enzalutamide is provided as liquid-filled soft gelatin capsules for oral administration. Each capsule contains 40 mg of enzalutamide as a solution in caprylocaproyl polyoxylglycerides. The inactive ingredients are caprylocaproyl polyoxylglycerides, butylated hydroxyanisole, butylated hydroxytoluene, gelatin, sorbitol sorbitan solution, glycerin, purified water, titanium dioxide, and black iron oxide.

8.2.1. Clinical Pharmacology

Following oral administration of enzalutamide 160 mg daily (the current FDA approved dose) in patients with metastatic castration-resistant prostate cancer, the median time to reach maximum plasma enzalutamide concentrations (Cmax) is 1 hour (range 0.5 to 3 hours). At steady state, the plasma mean Cmax values for enzalutamide and N-desmethyl enzalutamide (enzalutamide's major active metabolit) are 16.6 μg/mL (23% CV) and 12.7 μg/mL (30% CV),

respectively, and the plasma mean predose trough values are 11.4 µg/mL (26% CV) and 13.0 µg/mL (30% CV), respectively. The mean apparent volume of distribution (V/F) of enzalutamide in patients after a single oral dose is 110 L (29% CV).

Enzalutamide is 97% to 98% bound to plasma proteins, primarily albumin. N-desmethyl enzalutamide is 95% bound to plasma proteins. The mean apparent clearance (CL/F) of enzalutamide in patients after a single oral dose is 0.56 L/h (range 0.33 to 1.02 L/h). The mean terminal half-life (t1/2) for enzalutamide in patients after a single oral dose is 5.8 days (range 2.8 to 10.2 days). Following a single 160 mg oral dose of enzalutamide in healthy volunteers, the mean terminal t1/2 for N-desmethyl enzalutamide is approximately 7.8 to 8.6 days. In healthy volunteers, a high-fat meal did not alter the AUC to enzalutamide or N-desmethyl enzalutamide.

In vitro, human CYP2C8 and CYP3A4 are responsible for the metabolism of enzalutamide. Based on *in vivo* and *in vitro* data, CYP2C8 is primarily responsible for the formation of the active metabolite (N-desmethyl enzalutamide). Enzalutamide is primarily eliminated by hepatic metabolism. Following single oral administration of ^{14}C-enzalutamide 160 mg, 85% of the radioactivity is recovered by 77 days post dose: 71% is recovered in urine (including only trace amounts of enzalutamide and N-desmethyl enzalutamide), and 14% is recovered in feces (0.4% of dose as unchanged enzalutamide and 1% as N-desmethyl enzalutamide).

With the daily dosing regimen, enzalutamide steady state is achieved by Day 28, and enzalutamide accumulates approximately 8.3-fold relative to a single dose. Daily fluctuations in enzalutamide plasma concentrations are low (mean peak-to-trough ratio of 1.25). At steady state, enzalutamide showed approximately dose proportional pharmacokinetics over the daily dose range of 30 to 360 mg.

8.2.2. Safety/Precautions

- **Seizures:** In the Phase III post-docetaxel randomized clinical trial, 7 of 800 (0.9%) patients treated with enzalutamide 160 mg once daily experienced a seizure. No seizures occurred in patients treated with placebo. Patients experiencing seizure were permanently discontinued from therapy and all seizures resolved. There is no clinical trial experience re-administering enzalutamide to patients who experienced seizures.

  The safety of enzalutamide in patients with predisposing factors for seizure is not known because these patients were excluded from the trial. These exclusion criteria included a history of seizure, underlying brain injury with loss of consciousness, transient ischemic attack within the past 12 months, cerebral vascular accident, brain metastases,

brain arteriovenous malformation or the use of concomitant medications that may lower the seizure threshold.

Because of the risk of seizure associated with XTANDI use, patients should be advised of the risk of engaging in any activity where sudden loss of consciousness could cause serious harm to themselves or others.

- **Laboratory abnormalities:** In the randomized Phase III post-docetaxel trial, Grade 1-4 neutropenia occurred in 15% of patients on enzalutamide (1% Grade 3-4) and in 6% of patients on placebo (no Grade 3-4). The incidence of Grade 1-4 thrombocytopenia was similar in both arms; 0.5% of patients on enzalutamide and 1% on placebo experienced Grade 3-4 thrombocytopenia. Grade 1-4 elevations in ALT occurred in 10% of patients on enzalutamide (0.3% Grade 3-4) and 18% of patients on placebo (0.5% Grade 3-4). Grade 1-4 elevations in bilirubin occurred in 3% of patients on enzalutamide and 2% of patients on placebo.
- **Infections:** In the randomized Phase III post-docetaxel trial, 1.0% of patients treated with enzalutamide compared to 0.3% of patients on placebo died from infections or sepsis. Infection-related serious adverse events were reported in approximately 6% of the patients on both treatment arms.
- **Falls and fall-related injuries:** In the randomized Phase III post-docetaxel trial, falls or injuries related to falls occurred in 4.6% of patients treated with enzalutamide compared to 1.3% of patients on placebo. Falls were not associated with loss of consciousness or seizure. Fall-related injuries were more severe in patients treated with enzalutamide and included non-pathologic fractures, joint injuries, and hematomas.
- **Hallucinations:** In the randomized Phase III post-docetaxel trial, 1.6% of patients treated with enzalutamide were reported to have Grade 1 or 2 hallucinations compared to 0.3% of patients on placebo. Of the patients with hallucinations, the majority were on opioid-containing medications at the time of the event. Hallucinations were visual, tactile, or undefined.
- **Unforeseeable risks to embryo or fetus:** Enzalutamide is contraindicated in women. In theory, enzalutamide can cause fetal harm if administered to a pregnant woman based on its mechanism of action. While there are no human or animal data on the use of enzalutamide in pregnancy and enzalutamide is not indicated for use in women, it is important to know that maternal use of an androgen receptor inhibitor could affect

development of the fetus. Women who are pregnant or women who may be pregnant should not handle enzalutamide without protection, e.g., gloves. Patients should also be informed that it is not known whether enzalutamide or its metabolites are present in semen and they should use a condom if having sex with a pregnant woman. The patient should use a condom and another effective method of birth control if he is having sex with a woman of child-bearing potential. These measures are required during and for three months after treatment with enzalutamide.

8.2.3. Information for Patients

- Instruct patients to take their dose at the same time each day (once daily). enzalutamide can be taken with or without food. Each capsule should be swallowed whole. Do not chew, dissolve, or open the capsules.
- Inform patients that enzalutamide has been associated with an increased risk of seizure. Discuss conditions that may predispose to seizures and medications that may lower the seizure threshold. Advise patients of the risk of engaging in any activity where sudden loss of consciousness could cause serious harm to themselves or others.
- Inform patients that enzalutamide may cause dizziness, mental impairment, paresthesia, hypoesthesia, and falls.
- Inform patients that if they miss a dose, then they should take it as soon as they remember. If they forget to take the dose for the whole day, then they should take their normal dose the next day. They should not take more than 160 mg of enzalutamide per day.
- Apprise patients of the common side effects associated with enzalutamide. Direct the patient to a complete list of adverse drug reactions in the FDA-approved patient labeling (PATIENT INFORMATION).
- Inform patients that enzalutamide may be harmful to a developing fetus. Patients should also be informed that they should use a condom if having sex with a pregnant woman. A condom and another effective method of birth control should be used if the patient is having sex with a woman of child-bearing potential. These measures are required during and for three months after treatment with enzalutamide.

8.2.4. Laboratory tests

- Liver function tests (e.g. AST/ALT, bilirubin) should be monitored periodically while on enzalutamide.
- Complete blood counts (e.g. white blood cell count, hemoglobin, hematocrit and platelet count) should be monitored periodically while on enzalutamide.

8.2.5. Drug Interactions

Co-administration of a strong CYP2C8 inhibitor (gemfibrozil) increased the composite area under the plasma concentration-time curve (AUC) of enzalutamide plus N-desmethyl enzalutamide in healthy volunteers. Co-administration of enzalutamide with strong CYP2C8 inhibitors should be avoided if possible. If co-administration of enzalutamide with a strong CYP2C8 inhibitor cannot be avoided, reduce the dose of enzalutamide.

The effects of CYP2C8 inducers on the pharmacokinetics of enzalutamide have not been evaluated *in vivo*. Co-administration of enzalutamide with strong or moderate CYP2C8 inducers (e.g., rifampin) may alter the plasma exposure of enzalutamide and should be avoided if possible. Selection of a concomitant medication with no or minimal CYP2C8 induction potential is recommended.

Co-administration of a strong CYP3A4 inhibitor (itraconazole) increased the composite AUC of enzalutamide plus Ndesmethyl enzalutamide by 1.3 fold in healthy volunteers.

The effects of CYP3A4 inducers on the pharmacokinetics of enzalutamide have not been evaluated *in vivo*. Co-administration of enzalutamide with strong CYP3A4 inducers (e.g., carbamazepine, phenobarbital, phenytoin, rifabutin, rifampin, rifapentine) may decrease the plasma exposure of enzalutamide and should be avoided if possible. Selection of a concomitant medication with no or minimal CYP3A4 induction potential is recommended. Moderate CYP3A4 inducers (e.g., bosentan, efavirenz, etravirine, modafinil, nafcillin) and St. John's Wort may also reduce the plasma exposure of enzalutamide and should be avoided if possible.

Enzalutamide is a strong CYP3A4 inducer and a moderate CYP2C9 and CYP2C19 inducer in humans. At steady state, enzalutamide reduced the plasma exposure to midazolam (CYP3A4 substrate), warfarin (CYP2C9 substrate), and omeprazole (CYP2C19 substrate). Concomitant use of enzalutamide with narrow therapeutic index drugs that are metabolized by CYP3A4 (e.g., alfentanil, cyclosporine, dihydroergotamine, ergotamine, fentanyl, pimozide, quinidine, sirolimus and tacrolimus), CYP2C9 (e.g., phenytoin, warfarin) and CYP2C19 (e.g., S-mephenytoin)

should be avoided, as enzalutamide may decrease their exposure. If co-administration with warfarin cannot be avoided, conduct additional INR monitoring.

For more detail on specific drug interactions, please refer to the FDA prescribing information.

8.2.6. Adverse Reactions

The most common adverse drug reactions (≥ 5%) reported in patients receiving enzalutamide in the randomized clinical trial were asthenia/fatigue, back pain, diarrhea, arthralgia, hot flush, peripheral edema, musculoskeletal pain, headache, upper respiratory infection, muscular weakness, dizziness, insomnia, lower respiratory infection, spinal cord compression and cauda equina syndrome, hematuria, paresthesia, anxiety, and hypertension. Grade 3 and higher adverse reactions were reported among 47% of enzalutamide -treated patients and 53% of placebo-treated patients. Discontinuations due to adverse events were reported for 16% of enzalutamide -treated patients and 18% of placebo-treated patients. The most common adverse reaction leading to treatment discontinuation was seizure, which occurred in 0.9% of the enzalutamide -treated patients compared to none (0%) of the placebo-treated patients. Table 2 summarizes the adverse reactions reported in the randomized Phase II clinical trials.

8.2.7. Administration, Supply and Storage

Enzalutamide is marketed and approved for the indication under study.

8.2.7.1. Administration

Enzalutamide 160 mg daily (the current FDA approved dose), or four 40 mg capsules by mouth daily, will be administered.

8.2.7.2. Supply

Enzalutamide, marketed as Xtandi, comes in 40 mg capsules and are supplied as white to off-white oblong soft gelatin capsules imprinted in black ink with MDV. Enzalutamide capsules are available in bottles of 120 capsules (NDC 0469-0125-99).

8.2.7.3. Storage

Store enzalutamide capsules at 20°C to 25°C (68°F to 77°F) in a dry place and keep the container tightly closed. Excursions permitted from 15°C to 30°C (59°F to 86°F).

9. Data Monitoring and Reporting Requirements

Data Monitoring of this protocol will occur on a regular basis with the frequency dependent on the rate of subject accrual and the progress of the study. The protocol will be monitored internally at University of Washington/Fred Hutchinson Cancer Research Center (UW/FHCRC) by the Principal Investigator and in accordance with UW/FHCRC guidelines.

Additionally, scheduled meetings will take place weekly and will include the protocol principal investigator, research nurse, data manager, and, when appropriate, the collaborators, sub-investigators, and biostatistician involved with the conduct of the protocol.

During these meetings the investigators will discuss matters related to: safety of protocol participants, validity and integrity of the data, enrollment rate relative to expectation, characteristics of participants, retention of participants, adherence to protocol (potential or real protocol violations), data completeness, and progress of data for secondary objectives.

9.1. Lead Site Principal Investigator

The Principal Investigator, Michael Schweizer, MD, is responsible for performing the following tasks:

- Coordinating, developing, submitting, and obtaining approval for the protocol as well as its subsequent amendments
- Assuring that all participating institutions are using the correct version of the protocol
- Taking responsibility for the overall conduct of the study at all participating institutions and for monitoring the progress of the study
- Reviewing and ensuring reporting of Serious Adverse Events (SAEs)
- Reviewing data from all sites

9.2. Definitions

<u>Adverse Event (AE)</u>

Any untoward medical occurrence in a patient or clinical investigation subject administered a medicinal product and which does not necessarily have to have a causal relationship with this treatment. An AE can therefore be any unfavorable and unintended sign (including an abnormal laboratory finding), symptom, or disease temporally associated with the use of a medicinal product, whether or not related

to the medicinal product.

Adverse Events of Special Interest

Events that are actively monitoring as a result of a previously identified signal (even if non- serious), and are typically defined in the Protocol.

Adverse Drug Reaction (ADR)

A noxious and unintended response to any dose of the drug (or biological) product for which there is a reasonable possibility that the product cause the response. ‘‘Reasonable possibility’’ means there is evidence to suggest a causal relationship between the drug and the adverse event. Suspected adverse reaction implies a lesser degree of certainty about causality than adverse reaction, which means any adverse event caused by a drug.

Product Quality Complaint (PQC)

Any discrete concern that questions the identity, quality, durability, reliability, safety, efficacy or intended performance of a drug product. A complaint may allege an injury or malfunction associated with the use of the drug product. It may also involve the design, literature, packaging, advertising, availability, physical appearance or promotion of the drug product.

Serious Adverse Event (SAE)

A SAE is any sign, symptom or medical condition that emerges during treatment or during a post-treatment follow-up period that (1) was not present at the start of treatment and is not a chronic condition that was part of the patient’s medical history OR (2) was present at the start of treatment or as part of the patient’s medical history but worsened in severity and/or frequency during therapy, AND that meets any of the following regulatory criteria:

- Is fatal (i.e., results in death from any cause at any time) or life-threatening (i.e., the patient was, in the view of the investigator, at immediate risk of death from the reaction as it occurred)
- Required or prolonged hospitalization (see exclusions below)
- Results in persistent or significant disability/incapacity
- Constitutes a congenital anomaly or a birth defect
- Is medically significant, may jeopardize the subject and may require medical or surgical

intervention to prevent one of the outcomes listed above.

Events not considered to be serious adverse event are hospitalizations for the:

- *Routine treatment or monitoring of the studied indication, not associated with any deterioration in condition*
- *Treatment, which was elective or pre-planned, for a pre-existing condition that did not worsen*

Any serious adverse event occurring in a patient from the first day of treatment and until 4 weeks after the last dose of treatment must be reported. The period after discontinuing study drug may be extended if there is a strong suspicion that the drug has not yet been eliminated.

All serious adverse events must be followed to resolution (≤ 1 or baseline) or until considered stable or irreversible.

9.3. Evaluating Adverse Events

The grade and severity of the event will be determined using the DCT/NCI Common Terminology Criteria, CTCAE v.4.0. A copy of the CTCAE version 4.0 can be downloaded from the CTEP home page (http://ctep.cancer.gov). All appropriate treatment areas should have access to a copy of the CTCAE version 4.0. Study staff must use one of the CTCAE criteria to define the event. Adverse events not included in the CTCAE v.4.0 should be reported and graded under the “Other” adverse event within the appropriate category and grade 1 to 5 according to the general grade definitions, mild, moderate, severe, life- threatening, fatal or disabling, as provided in the CTCAE.

The event will be determined to be expected or unexpected

The determination of whether an AE is expected is based on agent-specific adverse event information provided in Section 7and 8 Pharmaceutical Information. Unexpected AEs are those not listed in the agent-specific adverse event information provided in Section 8 Pharmaceutical Information.

The event will be evaluated for relationship to the medical treatment or procedure. The Investigator should document his/her opinion of the relationship of the event to study medication as follows:

- *Unrelated*- The adverse event is clearly not related to the investigational agent(s).
- *Unlikely*- The adverse event is doubtfully related to the investigational agent(s).
- *Possible*-The adverse event may be related to the investigational agent(s).

- *Probable*-The adverse event is most likely related to the investigational agent(s).
- *Definite*- The adverse event is clearly related to the investigational agent(s). Based on this information, a decision will be made by the IND sponsor-investigator (Michael Schweizer) as to whether any serious adverse event should be reported to the FDA as an expedited 7- or 15-day report per 21 CFR 312.32 or reported with the IND annual report per 21 CFR 312.33. Any adverse event meeting the IRB of record's expedited reporting requirements should be submitted to the local IRB by the site PI accordingly.

9.4. Documenting Adverse Events

Each individual sign or symptom must be documented separately. Worksheets must be signed and dated by person conducting evaluation to be used as source documentation.The attribution of all adverse events must be verified by an investigator. Evaluation of laboratory toxicities may be documented directly on a printed laboratory report or CRF provided it is signed by the investigator. However, if an action was conducted due to this abnormality (e.g. RBC transfusion due to low Hgb) this would be recorded on the AE form also.

Recording should be done in a concise manner using standard, acceptable medical terms.

The adverse event recorded should not be a procedure or a clinical measurement (i.e. a laboratory value or vital sign) but should reflect the reason for the procedure or the diagnosis based on the abnormal measurement.

Preexisting conditions that worsen in severity or frequency during the Study should also be recorded (a preexisting condition that does not worsen is not an adverse event).

Further, a procedure or surgery is not an adverse event; rather, the event leading to the procedure or surgery is considered an adverse event. Any event requiring in-patient hospitalization that occurs during the course of a subject's participation in a trial must be reported as an SAE. Hospitalizations that do not meet the criteria for SAE reporting are:

- Reasons described in the Protocol, e.g. drug administration, Protocol-required testing
- Surgery or procedure planned prior to entry into the Study.

If, in the Sponsor Investigator's judgment, a clinical significant worsening from baseline is observed in any laboratory or other test parameter (e.g. electrocardiogram (ECG), angiogram), physical exam finding, or vital sign, a corresponding clinical adverse event should be recorded.

If a specific medical diagnosis has been made, that diagnosis or syndrome should be recorded as the adverse event, whenever possible. However, a complete description of the signs, symptoms and investigations which led to the diagnosis should be provided. For example, if clinically significant elevations of liver function tests are known to be secondary to hepatitis, "hepatitis" and not "elevated liver function tests" should be recorded. If the cause is not known, the abnormal test or finding should be recorded as an adverse event, using appropriate medical terminology (e/g/ thrombocytopenia, peripheral edema, QT prolongation).

9.5. Adverse Event Monitoring and Reporting

The PI and/or the research nurse will monitor each patient closely for the development of adverse events and toxicities and record all such events. The timely reporting of adverse events (including toxic deaths) is required by the Food and Drug Administration (FDA).

All serious adverse events that occur after the subject has signed the informed consent form or during the study must be reported to the Cancer Consortium IRB (CC-IRB, fax 206-667-6831). AEs that are *unexpected, possibly related to the study drug,* and *serious or suggest a risk of greater harm from the research then previously known* will be reported to the IRB within 10 calendar days of the Investigator's awareness of the event.

The Adverse Event Reporting Form is available online:
http://extranet.fhcrc.org/EN/sections/iro/irb/forms/index.html#Reporting (as of Jan2012).

SUSARs will be reported on the Expedited Reporting Form for Unanticipated Problems or Noncompliance. Special Reporting Situations (SRS) will be reported to CC-IRB either immediately or at annual renewal, upon consultation with IRB staff. The SAE, SUSAR or SRS should be recorded on the appropriate case report form (CRF).

Reports of SAEs should be signed and dated by the principal investigator. In the absence of the PI, reports should be signed and dated by the individual reporting the event. If s/he is not medically licensed, the report should also be signed by a licensed medical practitioner, preferably a sub-investigator for this protocol. The PI will review and sign the report at the next opportunity.

Each report should contain the following information:

- Protocol number
- Subject number
- Disease/histology, if applicable
- Date the event occurred
- Description of the SAE
- Relationship of the event to treatment or other causality
- Whether the event was "expected'
- Severity of the event
- Intervention
- Outcome of the event
- Detailed text that includes the following information:
- An explanation of how the SAE was handled
- A description of the patient's condition
- Indication whether the subject remains on study
- Recommendation whether an amendment will need to be made to the protocol and/or the consent form.

Relevant, redacted medical records should be provided as soon as they become available; autopsy reports should be provided for deaths if available. Determination of expectedness will be based on the contents of the current Investigator's Brochure or package insert.

If a subject is permanently withdrawn from the study because of a serious adverse event, this information must be included in the End of Study Case Report Form as well as all SAE/SUSAR reports.

9.6. Withdrawal from Study

The investigator may withdraw a patient from any phase of the study for any of the following reasons:

- Discontinuation of treatment criteria as defined in Section 6.8
- Sustained Side Effects: Patients who have sustained toxicities, such as hyperglycemia or hypertension that do not return to NCI CTCAE (version 4.0) Grade 1 or less with appropriate medical management, should be discontinued from the study treatment.
- Administration of prohibited medications: The patient will be discontinued from the protocol treatment when prohibited drug is administered. Supportive care medications are permitted with their use following institutional guidelines. The concurrent administration of other anticancer therapy, or immunotherapy is prohibited during study treatment Phase. Use of other investigational drug therapy for any reason is prohibited.
- Patient withdraws consent. In this event, the reason(s) for withdrawal must be documented and clarification if withdrawal of consent includes follow-up phase for progression data collection. A patient's decision to take part in the study is voluntary and he may choose not to take part in the study or to stop taking part at any time. If he chooses not to take part or to stop at any time, it will not affect his future medical care or medical benefits.

If a subject terminates the study early, an Early Termination visit will be performed.

10. Statistical Methods

Anticipating low toxicity for niclosamide, we propose an alternative to the standard 3+3 dose escalation trial design; we will use the continual reassessment method (CRM) to evaluate dose-related toxicities and to determine the recommended phase 2 dose.[44] If the expected low toxicity holds, relative to a 3+3 design, the CRM will escalate to higher dose levels faster, treat more patients at higher dose levels, and provide greater power for determining whether the steady-state serum concentration exceeds levels shown to inhibit prostate cancer cell lines. If dose-limiting toxicities accumulate faster than anticipated, dose levels will de-escalate accordingly.[46] Adverse events will be documented by incidence and severity graded according to the National Cancer Institute – Common Terminology Criteria for Adverse Events (CTCAE) version 4.0.

We will target a maximum 30% of patients incurring dose-limiting toxicities up to 30 days after the final (day 28) dose. Because we anticipate low toxicity, however, we do not expect to achieve this maximum toxicity, and consequently we pre-specify stopping the trial after accruing a maximum of 12 patients. Per the CRM, the first 3 patients will receive niclosamide 500 mg PO TID (i.e. Cohort 1). If there are sufficiently few DLTs, the next 3 patients will receive niclosamide 1000 mg PO TID (i.e. Cohort 2). If there are sufficiently few DLTs, the next 3 patients will receive niclosamide 1500 mg PO TID (i.e. Cohort 3). If there are sufficiently few DLTs, the last 3 patients will also enroll to Cohort 3. Dose escalation and de-escalation will dictated by the CRM and based on posterior probabilities determined by: 1) the assumed dose-toxicity model, which will be a 1-parameter power model with a Gamma(1,1) prior distribution, 2) assumed prior probabilities of DLTs of 5%, 10%, and 15% for Cohorts 1-3, respectively, 3) a target DLT rate of ≤30%, and 4) accumulating toxicity data from current and previous cohorts.

For comparison with the 3+3 design, we use a 1-parameter power model for the dose-toxicity relationship with a Gamma(1,1) prior distribution. The table below shows 3 dose-toxicity scenarios and corresponding frequencies of patients who receive each dose level under the CRM and 3+3 designs.[47] The CRM consistently assigns a higher frequency of patients to the highest dose. Note that in all scenarios the risk of dose-limiting toxicities at the highest dose does not exceed the maximum toxicity level of 30%.

	Dose level (PO TID)		
	500 mg	1000 mg	1500 mg
**Scenario 1**			
Actual risk	5%	5%	5%
CRM (3+3) frequency	27% (35%)	25% (33%)	48% (32%)
**Scenario 2**			
Actual risk	5%	10%	15%
CRM (3+3) frequency	27% (34%)	26% (35%)	48% (31%)
**Scenario 3**			
Actual risk	5%	20%	30%
CRM (3+3) frequency	28% (38%)	31% (39%)	41% (23%)

Table 2: Dose-toxicity Scenarios. Prior guesses of risks of dose-limiting toxicities and comparison of frequencies of patients receiving each dose level in CRM and 3+3 designs under 3 scenarios.

To examine statistical power for the secondary PK/PD endpoint, motivated by Andrews et al., we assume niclosamide steady-state concentration follows a normal distribution with mean 2.9 and standard deviation 1.4 μg/mL.[27] Allowing a 5% probability of a type 1 error, N=12 patients yields over 99% power to conclude that the mean steady-state concentration exceeds 0.25 μg/mL based on a 1-sided 1-sample t-test. If the mean steady-state concentration is lower, the variance is greater, or the distribution is positively skewed, power will be smaller. Under a wide range of possibilities, however, N=12 patients will provide sufficient power to test whether the mean steady-state concentration exceeds this target.

Mean niclosamide concentration *vs.* time will be plotted for each dose cohort. Pharmacokinetic parameters (i.e. C_{max}, C_{min}, C_{ss} and $t_{1/2}$) will be reported as means for each dose cohort along with the observed ranges. The percent change in PSA following 28-days of combination therapy will be presented as a waterfall plot, with the rate of PSA response (i.e. ≥50% decline in PSA from baseline) reported as percentages with 95% confidence intervals.

11. References

1. Beer TM, Armstrong AJ, Rathkopf DE, et al. Enzalutamide in Metastatic Prostate Cancer before Chemotherapy. The New England journal of medicine 2014.
2. de Bono JS, Logothetis CJ, Molina A, et al. Abiraterone and increased survival in metastatic prostate cancer. The New England journal of medicine 2011;364:1995-2005.
3. Ryan CJ, Smith MR, de Bono JS, et al. Abiraterone in metastatic prostate cancer without previous chemotherapy. The New England journal of medicine 2013;368:138-48.
4. Scher HI, Fizazi K, Saad F, et al. Increased survival with enzalutamide in prostate cancer after chemotherapy. The New England journal of medicine 2012;367:1187-97.
5. Antonarakis ES, Lu C, Wang H, et al. AR-V7 and resistance to enzalutamide and abiraterone in prostate cancer. The New England journal of medicine 2014;371:1028-38.
6. Asangani IA, Dommeti VL, Wang X, et al. Therapeutic targeting of BET bromodomain proteins in castration-resistant prostate cancer. Nature 2014;510:278-82.
7. Carreira S, Romanel A, Goodall J, et al. Tumor clone dynamics in lethal prostate cancer. Science translational medicine 2014;6:254ra125.
8. Chang KH, Li R, Kuri B, et al. A gain-of-function mutation in DHT synthesis in castration-resistant prostate cancer. Cell 2013;154:1074-84.
9. Chen CD, Welsbie DS, Tran C, et al. Molecular determinants of resistance to antiandrogen therapy. Nature medicine 2004;10:33-9.
10. Cho E, Montgomery RB, Mostaghel EA. Minireview: SLCO and ABC transporters: a role for steroid transport in prostate cancer progression. Endocrinology 2014;155:4124-32.
11. Drake JM, Graham NA, Lee JK, et al. Metastatic castration-resistant prostate cancer reveals intrapatient similarity and interpatient heterogeneity of therapeutic kinase targets. Proceedings of the National Academy of Sciences of the United States of America 2013;110:E4762-9.
12. Evaul K, Li R, Papari-Zareei M, Auchus RJ, Sharifi N. 3beta-hydroxysteroid dehydrogenase is a possible pharmacological target in the treatment of castration-resistant prostate cancer. Endocrinology 2010;151:3514-20.
13. Li Z, Bishop AC, Alyamani M, et al. Conversion of abiraterone to D4A drives anti-tumour activity in prostate cancer. Nature 2015;523:347-51.
14. Malik R, Khan AP, Asangani IA, et al. Targeting the MLL complex in castration-resistant prostate cancer. Nature medicine 2015;21:344-52.
15. Montgomery RB, Mostaghel EA, Vessella R, et al. Maintenance of intratumoral androgens in metastatic prostate cancer: a mechanism for castration-resistant tumor growth. Cancer research 2008;68:4447-54.
16. Mostaghel EA, Marck BT, Plymate SR, et al. Resistance to CYP17A1 inhibition with abiraterone in castration-resistant prostate cancer: induction of steroidogenesis and androgen receptor splice variants. Clinical cancer research : an official journal of the American Association for Cancer Research 2011;17:5913-25.
17. Mostaghel EA, Solomon KR, Pelton K, Freeman MR, Montgomery RB. Impact of circulating cholesterol levels on growth and intratumoral androgen concentration of prostate tumors. PloS one 2012;7:e30062.
18. Wright JL, Kwon EM, Ostrander EA, et al. Expression of SLCO transport genes in castration-resistant prostate cancer and impact of genetic variation in SLCO1B3 and SLCO2B1 on prostate cancer

outcomes. Cancer epidemiology, biomarkers & prevention : a publication of the American Association for Cancer Research, cosponsored by the American Society of Preventive Oncology 2011;20:619-27.
19. Yang M, Xie W, Mostaghel E, et al. SLCO2B1 and SLCO1B3 may determine time to progression for patients receiving androgen deprivation therapy for prostate cancer. Journal of clinical oncology : official journal of the American Society of Clinical Oncology 2011;29:2565-73.
20. Yeh S, Lin HK, Kang HY, Thin TH, Lin MF, Chang C. From HER2/Neu signal cascade to androgen receptor and its coactivators: a novel pathway by induction of androgen target genes through MAP kinase in prostate cancer cells. Proceedings of the National Academy of Sciences of the United States of America 1999;96:5458-63.
21. Yu Z, Chen S, Sowalsky AG, et al. Rapid induction of androgen receptor splice variants by androgen deprivation in prostate cancer. Clinical cancer research : an official journal of the American Association for Cancer Research 2014;20:1590-600.
22. Hu R, Denmeade SR, Luo J. Molecular processes leading to aberrant androgen receptor signaling and castration resistance in prostate cancer. Expert review of endocrinology & metabolism 2010;5:753-64.
23. Dehm SM, Schmidt LJ, Heemers HV, Vessella RL, Tindall DJ. Splicing of a novel androgen receptor exon generates a constitutively active androgen receptor that mediates prostate cancer therapy resistance. Cancer research 2008;68:5469-77.
24. Hu R, Dunn TA, Wei S, et al. Ligand-independent androgen receptor variants derived from splicing of cryptic exons signify hormone-refractory prostate cancer. Cancer research 2009;69:16-22.
25. Sun S, Sprenger CC, Vessella RL, et al. Castration resistance in human prostate cancer is conferred by a frequently occurring androgen receptor splice variant. The Journal of clinical investigation 2010;120:2715-30.
26. Nadiminty N, Tummala R, Liu C, et al. NF-kappaB2/p52 induces resistance to enzalutamide in prostate cancer: role of androgen receptor and its variants. Molecular cancer therapeutics 2013;12:1629-37.
27. Andrews P, Thyssen J, Lorke D. The biology and toxicology of molluscicides, Bayluscide. Pharmacology & therapeutics 1982;19:245-95.
28. Liu C, Lou W, Zhu Y, et al. Niclosamide inhibits androgen receptor variants expression and overcomes enzalutamide resistance in castration-resistant prostate cancer. Clinical cancer research : an official journal of the American Association for Cancer Research 2014;20:3198-210.
29. Liu C, Armstrong C, Zhu Y, Lou W, Gao AC. Niclosamide enhances abiraterone treatment via inhibition of androgen receptor variants in castration resistant prostate cancer. Oncotarget 2016.
30. Li Y, Li PK, Roberts MJ, Arend RC, Samant RS, Buchsbaum DJ. Multi-targeted therapy of cancer by niclosamide: A new application for an old drug. Cancer letters 2014;349:8-14.
31. Jin Y, Lu Z, Ding K, et al. Antineoplastic mechanisms of niclosamide in acute myelogenous leukemia stem cells: inactivation of the NF-kappaB pathway and generation of reactive oxygen species. Cancer research 2010;70:2516-27.
32. Courtney KD, Manola JB, Elfiky AA, et al. A Phase I Study of Everolimus and Docetaxel in Patients With Castration-Resistant Prostate Cancer. Clinical genitourinary cancer 2014.
33. Gao N, Zhang Z, Jiang BH, Shi X. Role of PI3K/AKT/mTOR signaling in the cell cycle progression of human prostate cancer. Biochemical and biophysical research communications 2003;310:1124-32.

34. Grunwald V, DeGraffenried L, Russel D, Friedrichs WE, Ray RB, Hidalgo M. Inhibitors of mTOR reverse doxorubicin resistance conferred by PTEN status in prostate cancer cells. Cancer research 2002;62:6141-5.
35. Majumder PK, Febbo PG, Bikoff R, et al. mTOR inhibition reverses Akt-dependent prostate intraepithelial neoplasia through regulation of apoptotic and HIF-1-dependent pathways. Nature medicine 2004;10:594-601.
36. Jain G, Cronauer MV, Schrader M, Moller P, Marienfeld RB. NF-kappaB signaling in prostate cancer: a promising therapeutic target? World journal of urology 2012;30:303-10.
37. Jin R, Yi Y, Yull FE, et al. NF-kappaB gene signature predicts prostate cancer progression. Cancer research 2014;74:2763-72.
38. Jin RJ, Lho Y, Connelly L, et al. The nuclear factor-kappaB pathway controls the progression of prostate cancer to androgen-independent growth. Cancer research 2008;68:6762-9.
39. Sun Y, Campisi J, Higano C, et al. Treatment-induced damage to the tumor microenvironment promotes prostate cancer therapy resistance through WNT16B. Nature medicine 2012;18:1359-68.
40. Balgi AD, Fonseca BD, Donohue E, et al. Screen for chemical modulators of autophagy reveals novel therapeutic inhibitors of mTORC1 signaling. PloS one 2009;4:e7124.
41. Wieland A, Trageser D, Gogolok S, et al. Anticancer effects of niclosamide in human glioblastoma. Clinical cancer research : an official journal of the American Association for Cancer Research 2013;19:4124-36.
42. Khanim FL, Merrick BA, Giles HV, et al. Redeployment-based drug screening identifies the anti-helminthic niclosamide as anti-myeloma therapy that also reduces free light chain production. Blood cancer journal 2011;1:e39.
43. Scher HI, Halabi S, Tannock I, et al. Design and end points of clinical trials for patients with progressive prostate cancer and castrate levels of testosterone: recommendations of the Prostate Cancer Clinical Trials Working Group. Journal of clinical oncology : official journal of the American Society of Clinical Oncology 2008;26:1148-59.
44. O'Quigley J, Pepe M, Fisher L. Continual reassessment method: a practical design for phase 1 clinical trials in cancer. Biometrics 1990;46:33-48.
45. Eisenhauer EA, Therasse P, Bogaerts J, et al. New response evaluation criteria in solid tumours: revised RECIST guideline (version 1.1). European journal of cancer (Oxford, England : 1990) 2009;45:228-47.
46. Hansen AR, Graham DM, Pond GR, Siu LL. Phase 1 trial design: is 3 + 3 the best? Cancer control : journal of the Moffitt Cancer Center 2014;21:200-8.
47. Sweeting M, Mander A, Sabin T. bcrm: Bayesian continual reassessment method designs for phase I dose-finding trials. Journal of Statistical Software 2013;54:1-26.
48. Siegel R, Ma J, Zou Z, Jemal A. Cancer statistics, 2014. CA: a cancer journal for clinicians 2014;64:9-29.
49. Huggins C, Hodges CV. Studies on prostatic cancer. I. The effect of castration, of estrogen and androgen injection on serum phosphatases in metastatic carcinoma of the prostate. Cancer research 1941;1:293-7.
50. Schweizer MT, Antonarakis ES. Abiraterone and other novel androgen-directed strategies for the treatment of prostate cancer: a new era of hormonal therapies is born. Therapeutic advances in urology 2012;4:167-78.

51. Roach M, 3rd, Thomas K. Overview of randomized controlled treatment trials for clinically localized prostate cancer: implications for active surveillance and the United States preventative task force report on screening? J Natl Cancer Inst Monogr 2012;2012:221-9.
52. Sweeney C, Chen Y, Carducci MA, et al. Impact on overall survival (OS) with chemohormonal therapy versus hormonal therapy for hormone-sensitive newly metastatic prostate cancer (mPrCa): An ECOG-led phase III randomized trial. 2014 Proceedings of the American Society of Clinical Oncology; 2014; Chicago, IL.
53. Leuprolide versus diethylstilbestrol for metastatic prostate cancer. The Leuprolide Study Group. The New England journal of medicine 1984;311:1281-6.
54. Scher HI, Heller G. Clinical states in prostate cancer: toward a dynamic model of disease progression. Urology 2000;55:323-7.
55. Mohler JL. Castration-recurrent prostate cancer is not androgen-independent. Adv Exp Med Biol 2008;617:223-34.
56. de Bono JS, Oudard S, Ozguroglu M, et al. Prednisone plus cabazitaxel or mitoxantrone for metastatic castration-resistant prostate cancer progressing after docetaxel treatment: a randomised open-label trial. Lancet 2010;376:1147-54.
57. Kantoff PW, Higano CS, Shore ND, et al. Sipuleucel-T immunotherapy for castration-resistant prostate cancer. The New England journal of medicine 2010;363:411-22.
58. Parker C, Nilsson S, Heinrich D, et al. Alpha emitter radium-223 and survival in metastatic prostate cancer. The New England journal of medicine 2013;369:213-23.
59. Scher HI, Beer TM, Higano CS, et al. Antitumour activity of MDV3100 in castration-resistant prostate cancer: a phase 1-2 study. Lancet 2010;375:1437-46.
60. Al-Hadiya BM. Niclosamide: comprehensive profile. Profiles of drug substances, excipients, and related methodology 2005;32:67-96.
61. WHO Model List of Essential Medicines. (Accessed February 24, 2015, 2015, at http://www.who.int/medicines/publications/essentialmedicines/en/.)
62. Data Sheet on Pesticides, No. 63, Niclosamide (WHO/VBC/DS/88.63). (Accessed February 24, 2015, 2015, at http://www.inchem.org/documents/pds/pds/pest63_e.htm.)
63. Hu R, Lu C, Mostaghel EA, et al. Distinct transcriptional programs mediated by the ligand-dependent full-length androgen receptor and its splice variants in castration-resistant prostate cancer. Cancer research 2012;72:3457-62.
64. Joseph JD, Lu N, Qian J, et al. A clinically relevant androgen receptor mutation confers resistance to second-generation antiandrogens enzalutamide and ARN-509. Cancer discovery 2013;3:1020-9.
65. Mehra R, Kumar-Sinha C, Shankar S, et al. Characterization of bone metastases from rapid autopsies of prostate cancer patients. Clinical cancer research : an official journal of the American Association for Cancer Research 2011;17:3924-32.
66. Taylor BS, Schultz N, Hieronymus H, et al. Integrative genomic profiling of human prostate cancer. Cancer cell 2010;18:11-22.
67. Antonarakis E, Lu C, Wang H. AR-V7 and resistance to enzalutamide and abiraterone in prostate cancer. New England Journal Medicine 2014;In press.
68. Antonarakis ES, Lu C, Luber B, et al. AR-V7 and efficacy of abiraterone (Abi) and enzalutamide (Enza) in castration-resistant prostate cancer (CRPC): Expanded analysis of the Johns Hopkins cohort. American Society of Clinical Oncology Annual Meeting; 2016; Chicago, IL.

69. Goodman and Gilman's The Pharmacological Basis of Therapeutics. 8th ed. New York, NY: Pergamon Press; 1990.
70. Bussy U, Chung-Davidson YW, Li K, Li W. Phase I and phase II reductive metabolism simulation of nitro aromatic xenobiotics with electrochemistry coupled with high resolution mass spectrometry. Analytical and bioanalytical chemistry 2014;406:7253-60.
71. PubChem: Niclosamide. (Accessed December 29, 2014, at http://pubchem.ncbi.nlm.nih.gov/compound/niclosamide - section=IUPAC-Name.)
